# A lower impact of an acute exposure to electronic cigarette aerosols than to cigarette smoke in human organotypic buccal and small airway cultures was demonstrated using systems toxicology assessment

**DOI:** 10.1007/s11739-019-02055-x

**Published:** 2019-03-05

**Authors:** Anita R. Iskandar, Filippo Zanetti, Athanasios Kondylis, Florian Martin, Patrice Leroy, Shoaib Majeed, Sandro Steiner, Yang Xiang, Laura Ortega Torres, Keyur Trivedi, Emmanuel Guedj, Celine Merg, Stefan Frentzel, Nikolai V. Ivanov, Utkarsh Doshi, Kyeonghee Monica Lee, Willie J. McKinney, Manuel C. Peitsch, Julia Hoeng

**Affiliations:** 1 Philip Morris International R&D, Philip Morris Products S.A., Quai Jeanrenaud 5, 2000 Neuchâtel, Switzerland; 2 Altria Client Services LLC, Richmond, VA 23219 USA

**Keywords:** E-cigarettes, Organotypic cultures, Systems toxicology, Transcriptomics, Oral, Small airway

## Abstract

In the context of tobacco harm-reduction strategy, the potential reduced impact of electronic cigarette (EC) exposure should be evaluated relative to the impact of cigarette smoke exposure. We conducted a series of in vitro studies to compare the biological impact of an acute exposure to aerosols of “test mix” (flavors, nicotine, and humectants), “base” (nicotine and humectants), and “carrier” (humectants) formulations using *MarkTen*^®^ EC devices with the impact of exposure to smoke of 3R4F reference cigarettes, at a matching puff number, using human organotypic air–liquid interface buccal and small airway cultures. We measured the concentrations of nicotine and carbonyls deposited in the exposure chamber after each exposure experiment. The deposited carbonyl concentrations were used as representative measures to assess the reduced exposure to potentially toxic volatile substances. We followed a systems toxicology approach whereby functional biological endpoints, such as histopathology and ciliary beating frequency, were complemented by multiplex and omics assays to measure secreted inflammatory proteins and whole-genome transcriptomes, respectively. Among the endpoints analyzed, the only parameters that showed a significant response to EC exposure were secretion of proteins and whole-genome transcriptomes. Based on the multiplex and omics analyzes, the cellular responses to EC aerosol exposure were tissue type-specific; however, those alterations were much smaller than those following cigarette smoke exposure, even when the EC aerosol exposure under the testing conditions resulted in a deposited nicotine concentration approximately 200 times that in saliva of EC users.

**Electronic supplementary material:**

The online version of this article (10.1007/s11739-019-02055-x) contains supplementary material, which is available to authorized users.

## Introduction

Cigarette craving hinders success in smoking cessation, but studies have shown that substituting cigarettes with other sources of nicotine can facilitate accomplishing smoking abstinence [1]. In contrast to cigarette smoke (CS), in which more than 6000 substances have been identified [2], EC does not contain tobacco but contain liquids, known as e-liquids (EL), which are aerosolized upon heating. The EL formulations generally comprise nicotine, flavors, and humectants [3, 4]. The humectants are solvent carriers, which are aerosol formers, such as propylene glycol and glycerol. Most flavorings in EC are designated as generally recognized as safe (GRAS) by the U.S. Food and Drug Administration under the intended use as food additives for ingestion, but not necessarily for inhalation [3].

Perceptions of potential risks and benefits of the use of electronic cigarettes (EC) vary greatly among users and the public health community. Recent clinical studies reported that EC use did not alter lung function but resulted in significant improvement of the chronic obstructive pulmonary disease (COPD) exacerbation rates and COPD Assessment Tool scores in COPD patients [5]. Song et al. reported that EC use did not lead to significant alterations in the inflammatory cell counts, and mRNA and microRNA gene expression despite increased cytokines in the urine [6]. Another clinical study reported that switching to EC significantly reduced diastolic blood pressure short-term although this effect was not significant after adjusting for other factors [7]. Nonetheless, the Royal College of Physicians stated that the harm of EC use is unlikely to exceed 5% of that from cigarette smoking [8]. The Public Health England released a statement that “the health risks posed by e-cigarettes are relatively small by comparison [than smoking]” [9]. Furthermore, the Committee on the Review of the Health Effects of Electronic Nicotine Delivery Systems under the National Academies of Sciences, Engineering, and Medicine concluded that nicotine exposure from EC use “will likely pose minimal cancer risk to users” but “likely elevates the cardiovascular disease risk in people with preexisting cardiovascular diseases” [3]. The committee also stated that “in vitro toxicological tests, and short-term human studies suggest that e-cigarettes are likely to be far less harmful than combustible cigarettes,” although the absolute risks of the product cannot be determined at present [3]. Understanding the health effects of EC, therefore, requires the determination of not only the risk of using them but also the biological effects relative to those of combustible tobacco cigarette use [3].

Numerous studies have evaluated the potential toxicity of ECs in vitro [10–14]; however, a large number of in vitro studies examined the effects of the EL that were applied directly on cell cultures but not the effects of exposure to the vapor/aerosol [15–22]. Although such studies may provide insights into the potential toxicity of the EL, they do not reflect the real exposure to EC; in reality, the human respiratory system is exposed to the EC aerosols and not to the EL. Therefore, it is pertinent to evaluate the effects of the heated and aerosolized EL, an approach we put forward previously [23]. When heated and aerosolized, chemical reactions may result in the formation of new compounds. For example, carbonyls, such as formaldehyde, acetaldehyde, and acrolein, have been detected in EC aerosols [3, 24]; these compounds can cause DNA damage and mutagenesis [3]. Furthermore, studying the potential toxicity of inhaled aerosol mixtures in vitro also requires a proper and accurate particle size characterization to better understand the measureable outcomes following exposure. Particle size is one important property of aerodynamic behavior that influence the deposition of the aerosol components [25]. It should be, however, acknowledged, that the human respiratory tract has a complex anatomical structure that cannot be easily simulated in vitro (i.e., the deposition of compounds in the in vitro exposure chamber cannot accurately reflect the regional deposition of compounds in the human lung).

To test the effect of whole aerosols/smoke, three-dimensional (3D) organotypic in vitro air–liquid interface (ALI) epithelial cell cultures are favorable. The ALI configuration exposes the apical side of the culture to ambient air. This configuration allows exposing the cultures to whole aerosol/smoke under a condition resembling that of smoking. Furthermore, compared with 2D culture systems, 3D cultures have been widely acknowledged to more accurately reflect the in vivo tissue environment from which cultured cells are derived [26].

Therefore, in this study, to understand the potential reduced risk of EC use in human oral and lung epithelia, we compared the impact of an acute exposure to undiluted EC aerosols with that of an acute exposure to diluted CS, at a matching puff number (112 puffs), using in vitro human 3D organotypic buccal epithelial and small airway epithelial cell cultures. The EC aerosols tested were generated from the prototype EL “test mix” (flavors, nicotine, and humectants), “base” (nicotine and humectants), and “carrier” (humectants only), using *MarkTen*^®^ EC devices. We first determined the particle sizes of the EC aerosols and CS. The concentrations of nicotine and carbonyls deposited in the exposure chamber were also measured after each exposure experiment. Subsequently, we assessed the potential reduced impact of EC exposure by conducting histological assessment and measuring ciliary beating frequency (CBF) relative to the impact of CS exposure (CBF was measured only in the ciliated small airway epithelial culture). Finally, we profiled the expression of inflammatory mediators secreted into the media and the transcriptome in the cultures to determine the underlying biological mechanisms triggered by exposure to EC aerosols and CS.

## Materials and methods

### Human organotypic buccal and small airway epithelial cultures

Organotypic human buccal epithelial (EpiOral™, MatTek Corp., Ashland, MA, USA) and small airway epithelial (SmallAir™, Epithelix. Geneva, Switzerland) cultures were used; each was reconstituted from the primary cells of a single donor (Table 1). A single donor was used to reduce the influence of donor-to-donor variability and hence to increase the statistical power to identify potential exposure effects, although the use of cells from a single donor can only indicate a donor-specific response. The cells were grown in Transwell^®^ inserts (with a 6.5-mm diameter, and a 0.4-μm pore size) and maintained in 24-well culture plates at the ALI at 37°C (5% CO_2_, 90% humidity) in their respective culture media (0.7 mL/well) according to the manufacturers’ instructions.

**Table 1:** Human buccal and small airway culture models

Description	EpiOral™	SmallAir™
Cell origin	Human buccal epithelial cells	Human bronchiolar epithelial cells
Culture model	Three-dimensional organotypic monoculture	Three-dimensional organotypic monoculture
Donor profile	46 years, male	65 years, male
Donor smoking status	Nonsmoker	Nonsmoker
Donor pathology status	No pathology reported	No pathology reported

The cultures were fully differentiated and acclimatized in the incubator before exposure. After exposure, the medium was not changed until it was collected for various endpoint measurements.

### Reference cigarette smoke and electronic cigarette aerosols

**Fig. 1:**
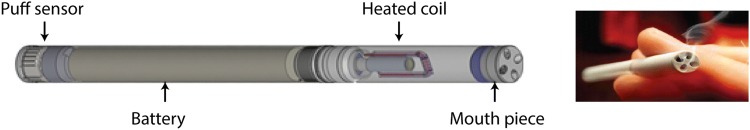
*MarkTen*^®^ devices. A cartridge of *MarkTen*^®^ contains 0.9 g of e-liquid. The puff activate product generates the aerosol via a 3.5 Ω (3.9 W) heater coil

Mainstream CS was generated from 3R4F reference cigarettes, purchased from the University of Kentucky, Lexington, KY, USA [27]. Test aerosols were generated from three different EL prototype formulations (Table 2), provided by Altria Client Services (ALCS) LLC, Richmond, VA, USA using *MarkTen*^®^ EC devices (ALCS), Fig. 1.

**Table 2:** Electronic cigarette liquid formulations

Description	Propylene glycol and glycerol	Nicotine	Flavor formulation
Test mix	✔	✔	✔
Base	✔	✔	NA
Carrier	✔	NA	NA

The nicotine concentration in the formulation was 4% by weight (w/w). *NA* not applicable

### Exposure setup

**Fig. 2:**
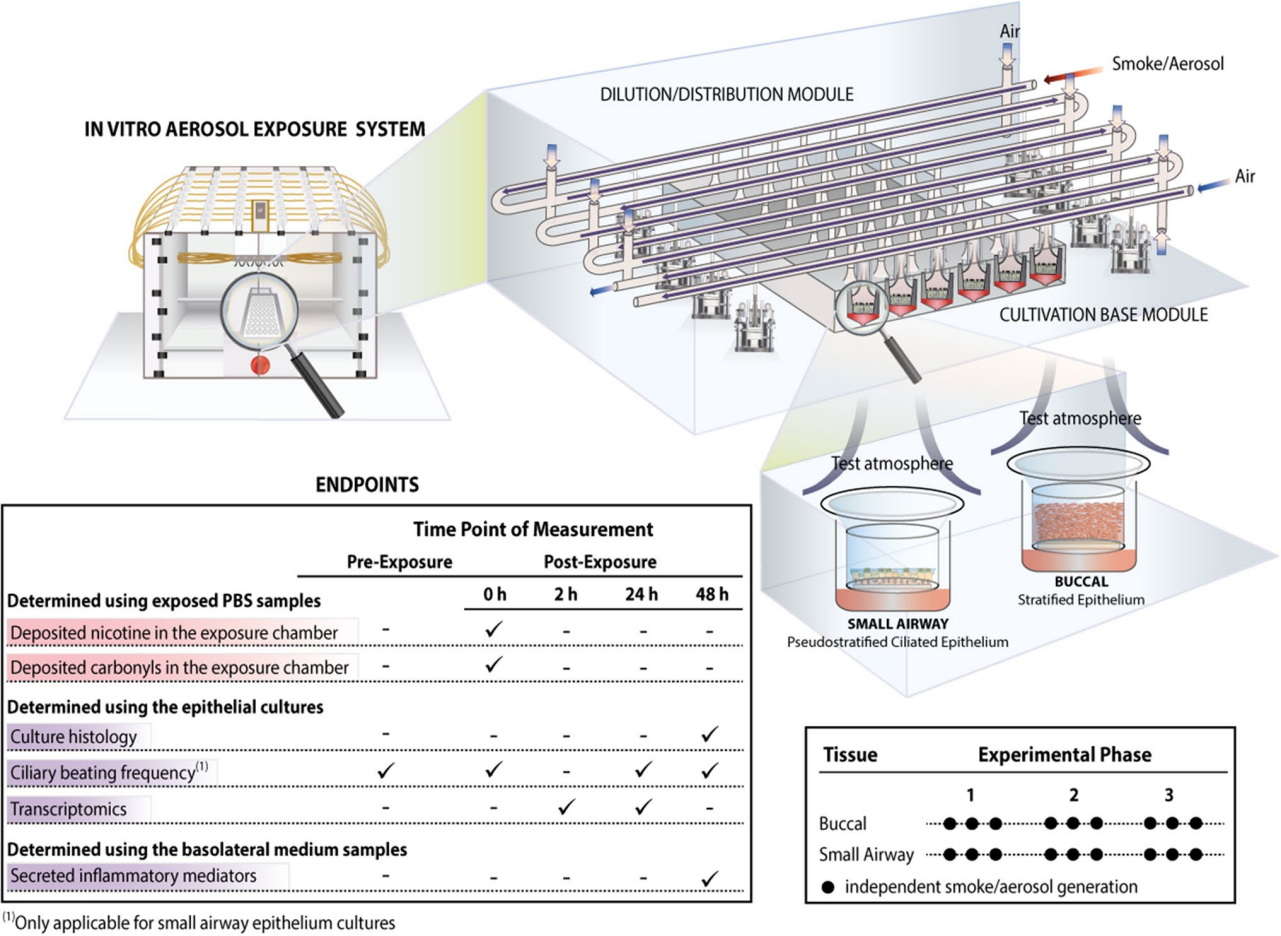
Experimental design and biological endpoints. Human organotypic buccal and small airway cultures were exposed in an in vitro aerosol exposure system (the Vitrocell^®^ 24/48 exposure systems is illustrated). Three experimental phases, each including three independent aerosol/smoke generations, were performed using new batches of buccal and small airway cultures, totaling nine repetitions per culture type. Various endpoints were measured at specific time points before and after exposure, from each smoke/aerosol generation. PBS, phosphate-buffered saline

The study consisted of three experimental phases using buccal cultures and three phases using small airway cultures (Fig. 2). In each phase, three independent smoke and aerosol generations were performed (paired with their corresponding air-exposure controls), totaling nine independent generations (i.e., data points). For each phase, a new batch of cultures was used; exposures were conducted separately for each culture type. Various endpoints were assessed following exposures (Fig. 2).

Two independent exposure systems (Vitrocell^®^ 24/48; Vitrocell Systems GmbH, Waldkirch, Germany) were used: one for 3R4F reference CS, and the other for EC aerosol exposures. The Vitrocell^®^ 24/48 exposure system is equipped with a Dilution/Distribution Module, into which fresh air can be added to dilute any aerosol serially in various rows (Fig. 2). During exposure, smoke or aerosol is passed through the Dilution/Distribution Module and distributed into the Cultivation Base Module via port ejectors (trumpets) by negative pressure [28]. The culture models were placed in the Cultivation Base Module and exposed to smoke or aerosol at their apical sides.

The aerosol generation from the EC aerosols was performed using single-programmable syringe pumps, which were connected to one dedicated Vitrocell^®^ 24/48 exposure system. For each EL formulation, two *MarkTen*^®^ devices were connected to one pump; a pinch valve—installed between the devices and the pump—alternated the activation of the two devices and resulted in the generation of one puff every 15 s (a total of 4 puffs/min was generated). Two devices (of the same formulation) were used simultaneously, in an alternate manner: a total of two devices was used during the 28-min exposure [one of the device was pre-puffed (57–112th puff were used), and the other was not (1st–56th puff were used)]. New devices were used for each aerosol generation (Fig. 2). Before being used for the experiment, the ECs were stored at room temperature in their original packaging.

The CS generation from the 3R4F cigarettes were performed using a 30-port carousel smoking machine (SM2000; Philip Morris, International), which was connected to another dedicated Vitrocell^®^ 24/48 exposure system. A total of 4 puffs/min were taken consecutively from two 3R4F cigarettes placed in the 30-port carousel (puff frequency was every 15 s). For the 28-min exposure, a total of ten 3R4F cigarettes were smoked. The 3R4F cigarettes were stored in their original packaging at 5 ± 3°C with uncontrolled humidity conditions. Before being used for the experiment, 3R4F cigarettes were conditioned for between 2–10 days under controlled conditions at 22 ± 1 °C and a relative humidity of 60 ± 3% according to ISO guideline 3402 [29].

Smoke and aerosol generation was conducted according to the parameters given in Table 3.

**Table 3:** Smoke and aerosol generation parameters

Item	Puff volume (mL)	Puff duration (s)	Frequency (puff per 60 s, per item)^a^	Puff exhaust time (s)	Puff count per item^a^	Puff profile
Test mix, base, carrier	55 (at the port of the e-cigarette aerosol collection machine)	5	2	8	Max of 140 puffs per *MarkTen*^®^ cartridge	Square shaped
3R4F reference cigarette^b^	55	2	2	8	10–12 puffs per cigarette	Bell shaped

^a^One item refers to one *MarkTen*^®^ EC cartridge for test mix, base, or carrier EL; or one stick of 3R4F reference cigarette

^b^Health Canada (HC) smoking regimen with ventilation holes blocked [30]

For the current study, a set of organotypic cultures was exposed to various concentrations of 3R4F CS and to air, simultaneously, in one exposure system (Table 4). For 3R4F CS, cultures were exposed for 28 min (112 puffs) at various smoke concentrations (diluted in air). The concentrations of 3R4F CS were selected based on the sensitivity of human organotypic cultures observed in previous studies [31–34], from which we detected a considerable range of biological effects from gene expression to morphology. Another set of cultures was exposed to undiluted test mix, base, and carrier EC aerosols and to air, simultaneously, in another exposure system (Table 4).

**Table 4:** Smoke and aerosol exposure doses

	Smoke or aerosol concentration (%) fed in to the exposure chamber^a^	Duration of exposure (min)	Total puff numbers	Median concentration of deposited nicotine in the exposure chamber (µg nicotine/mL PBS)
Buccal culture experiments				
Air (control for 3R4F)	0^b^	28	112	NA
3R4F concentration 1	13	28	112	18^c^
3R4F concentration 2	24	28	112	33
3R4F concentration 3	69	28	112	131
Air (control for EC aerosols)	0^b^	28	112	NA
Test mix (undiluted)	100	28	112	210^c^
Base (undiluted)	100	28	112	216^c^
Carrier (undiluted)	100	28	112	NA
Small airway culture experiments				
Air (control for 3R4F)	0^b^	28	112	NA
3R4F concentration 1	3	28	112	2
3R4F concentration 2	7	28	112	9
3R4F concentration 3	13	28	112	18^c^
Air (control for EC aerosols)	0^b^	28	112	NA
Test mix (undiluted)	100	28	112	210^c^
Base (undiluted)	100	28	112	216^c^
Carrier (undiluted)	100	28	112	NA

*PBS* phosphate-buffered saline, *NA* not applicable

^a^Values refer to the smoke or aerosol concentration fed to the Vitrocell^®^ 24/48 Dilution/Distribution Module

^b^Controls refer to 100% air administered to the culture on the same exposure plate

^c^Because identical doses were administered to both buccal and small airway cultures, the aggregated values (median concentrations) of deposited nicotine are indicated

### Analysis of particle size distribution

The particle size distribution of the test mix, base, and carrier aerosols, as well as that of 3R4F CS, was measured upstream (before going into the Vitrocell^®^ 24/48 exposure system) using the Aerodynamic Particle Sizer^®^ (APS™, model 3321; TSI Incorporated, Shoreview, MN, USA), which was connected directly to the outlet of the single-programmable syringe pumps. This closed connection was established using a 1-m conductive tube with a 1-cm inner diameter. A T-junction (opens to the surrounding environment) was installed upstream of the APS™ to avoid buildup of negative pressure inside the connection that was expected as a consequence of the spectrometer-generated flow (at a volume-flow-rate of 5 L/min). The aerosol was supplied actively (by the action of the syringe pumps); therefore, the APS™ extracted only the volume of surrounding air necessary to compensate for the difference between the aerosol volume-flow-rate and the volume-flow-rate generated by the instrument; this meant that the complete aerosol volume was subjected to analysis. The particle concentrations in the 3R4F CS or EC aerosols provided by the pump were expected to be outside the working range of the APS™; therefore, a 100-fold dilution was applied by installing the 3302A Aerosol Diluter (TSI Incorporated) upstream of the APS™.

### Analysis of nicotine in phosphate-buffered saline

Concentrations of the deposited nicotine in the exposure chamber were measured in the exposed PBS, which did not contain MgCl_2_ or CaCl_2_ (Sigma-Aldrich, St. Louis, MO, USA; Ref. D8357). One hundred microliters of PBS-filled steel inserts were located in the Base Module of the Vitrocell^®^ 24/48 exposure system and exposed together with the buccal or small airway epithelial cultures, in every exposure experiment. Concentrations of nicotine were measured using liquid chromatography tandem-mass spectrometry.

### Analysis of carbonyls in phosphate-buffered saline

The entire row of the Base Module of the Vitrocell^®^ 24/48 exposure system was filled with PBS and exposed together with the epithelial cultures, in every exposure experiment. Before exposure, each row in the Cultivation Base Module of the Vitrocell^®^ 24/48 exposure system was filled with 18.5 mL PBS. Following exposure, an aliquot of 1.2 mL PBS-exposed sample (per row) was collected and subjected to high-performance liquid chromatography coupled with tandem-mass spectrometry analysis, as previously reported [28].

### Histology

Histological samples were obtained only from cultures harvested 48 h post-exposure, as conducted in our previous studies [35, 36] showing that morphological alterations would occur at later time points after molecular changes took place [37]. The processing of the organotypic cultures followed a previously published protocol [32]. Briefly, cultures were fixed for 2 h in freshly prepared 4% paraformaldehyde, and then removed from the insert for paraffin embedding using the tissue processor Leica ASP300S (Leica Biosystems Nussloch GmbH, Nussloch, Germany). Sections of 5-μm thickness were obtained and mounted on glass slides, which were subsequently stained with hematoxylin (Merck Millipore, Billerica, MA, USA), eosin (Sigma-Aldrich), and Alcian blue (Sigma-Aldrich). Digital microscopic images were generated using the slide scanner Hamamatsu NanoZoomer 2.0 (Hamamatsu Photonics, K.K., Hamamatsu, Japan). Histological assessment was conducted by a trained independent certified pathologist (Unilabs Independent Histopathology Services, London, UK). The specification of the various histopathological findings that were assessed is given in Supplementary Table 1.

### Measurement of ciliary beating frequency

CBF measurement was conducted in small airway cultures only (not applicable for the nonciliated buccal cultures) using the Sisson Ammons Video Analysis system (Ammons Engineering, Clio, MI USA). Briefly, the ciliary beating videos were recorded using a video camera (Basler acA1300–200 µm; Basler AG, Ahrensburg, Germany) using a 4 × magnification (Leica DMi8 light microscope; Leica Microsystems, Heerbrugg, Switzerland) and following a set of parameters: a frame rate of 100 frames per second; a frame resolution of 640 by 480 pixels; a total number of 512 frames; and an 8-bit greyscale precision (256 levels of intensity). Ciliary beating of the cultures, on the field of interest selected from the center of each small airway culture, was measured before the exposure, immediately after exposure (0 h post-exposure), and 24 h and 48 h post-exposure. For the measurement, small airway cultures were transferred to a stage-top incubator (CU-501; Live Cell Instruments, Seoul, Korea). First, the data were processed by pixel, for which subsets of the pixels were processed individually. Their samplings were performed at regular steps, 1–8 on both directions (width and height), achieving an overall sampling ratio of 1 to 64. Then, the pixel-wise spectrum analysis was performed by: (1) centering the signal; (2) subtracting the mean of the pixel intensity over time (the 512 frames); (3) performing a fast Fourier transform on this signal, to estimate the periodogram power spectral density of this signal in the range from 0 to 50 Hz; and finally (4) smoothing the spectral density using penalized B-splines. Second, the data were processed by movies according to the following parameters: (1) the median spectral density was computed over the processed pixels by movies; (2) the median spectral density was smoothed using penalized B-splines; (3) the dominant frequency was determined as the frequency related to the highest spectral power in the range of expected cilia beating frequencies (2.5–25 Hz; the lower end, 2.5 Hz, was used to exclude high spectral power detected below 2.5 Hz, which was likely associated with movement of mucus above the culture that was visible in the video recording, while the upper end, 25 Hz, was used to exclude high-frequency noises often detected in severely damaged culture). The width of the peak containing the dominant frequency was taken as the frequency range of which the spectral power decreased monotonically from the peak; and (4) the weighted frequency was determined as the weighted mean of the peak frequencies (weighting the frequencies by their spectral power).

### Measurement of secreted inflammatory mediators

Multi-analyte profiling of inflammatory mediators secreted into the basolateral medium of cultures was performed using commercially available Milliplex panels (Merck Millipore) with Luminex^®^ xMAP^®^ Technology (Luminex, Austin, TX, USA)-based analysis according to the manufacturer’s instructions. Briefly, 25 µL of diluted and non-diluted sample was used for each detection and the analysis was run on the FLEXMAP 3D^®^ platform, equipped with xPONENT^®^ software version 4.2 (Luminex). Data were presented as Median Fluorescent Intensity using a five-parameter logistic or spline curve-fitting method to calculate the analyte concentrations in the basolateral medium samples. The following analytes (inflammatory mediators) were measured: chemokine [C–C motif] ligand (CCL) 20; chemokine [C–X–C motif] ligand (CXCL) 1 (also known as GRO alpha), CXCL-8 (also known as interleukin [IL]-8) and IL-6; tumor necrosis factor alpha (TNFα); soluble intercellular adhesion molecule (sICAM) 1; matrix metalloproteinase (MMP)-1 and MMP-9; tissue inhibitor of metalloproteinase (TIMP) 1, and vascular endothelial growth factor (VEGF) alpha. The data were log-transformed, and the geometric means were used to calculate the fold-changes of the mediators (exposed vs. air-exposed samples).

### Statistical analyzes

Basic descriptive statistical measures, such as mean, median, and standard deviation for all the investigated endpoints were computed (except for the analysis of mRNA data, see “Analysis of mRNA data” section). CS or EC aerosol-exposed groups were compared with the air-exposed controls using paired *t* test (paired within the same exposure run). The analyzes were performed in R-3.2.2 or R3.1.2, but the analyzes on ciliary beating was performed using SAS 9.2. Datasets, further detail on the protocols, and additional data visualizations are available on the INTERVALS platform at https://www.intervals.science/studies/#/MarkTen_Organotypic_PMI-Altria.

### RNA isolation and array analyzes

Total RNA was isolated from the epithelial cultures using a previously published method [32, 34]. Briefly, for the mRNA array, 100 ng of total RNA was reverse transcribed to cDNA using an Affymetrix^®^ HT 3′-IVT PLUS kit (Thermo Fisher Scientific, Waltham, MA, USA). The cDNA was labeled and amplified to complementary RNA (cRNA). The fragmented and labeled cRNA was hybridized to a GeneChip^®^ Human Genome U133 Plus 2.0 Array (Thermo Fisher Scientific) in a 645 GeneChip^®^ Hybridization Oven (Thermo Fisher Scientific) according to the manufacturer’s instructions. Arrays were rinsed and stained on a GeneChip^®^ FS450 DX Fluidics Station (Thermo Fisher Scientific) using the Affymetrix^®^ GeneChip^®^ Command Console^®^ Software (AGCC software v-3.2, protocol FS450_0001).

Finally, the arrays were scanned using a GeneChip^®^ Scanner 3000 7G (Thermo Fisher Scientific). Raw images from the scanner were saved as DAT files. The AGCC software automatically gridded each DAT file image and extracted probe cell intensities into an Affymetrix CEL file.

### Analysis of mRNA data

A model was fitted using *limma* R package [38] to estimate the treatment effect (for each experimental factor combination item, concentration and post-exposure duration) by including the covariate exposure run as a blocking variable to account for the pairing during an exposure run (exposed vs. air-exposed control samples). The *p*-values for each computed effect were adjusted across genes using the Benjamini–Hochberg false discovery rate (FDR) method [39]. Differentially expressed genes (DEGs) were defined as a set of genes whose FDR was < 0.05. The mRNA array datasets can be accessed in the Arrays Express repository (ID: E-MTAB-7577).

### Processing raw CEL files from the mRNA microarray analyzes

The raw CEL files were background corrected, normalized, and summarized using the frozen-robust multi-array analysis [40]. Background correction and quantile normalization were used to generate microarray expression values from all arrays passing quality controls and were performed using the custom chip definition files environment HGU133Plus2_Hs_ENTREZG v16.0 [41], as previously described in greater detail [31, 33, 36].

### Network-based enrichment analysis of transcriptomic data

Quantitative assessment of the transcriptomic data was conducted using a network enrichment approach and network perturbation amplitude (NPA) algorithm, described in greater detail in a previous publication [42]. Briefly, the methodology aims to contextualize transcriptome profiles (treated vs control, or aerosol/smoke-exposed vs air-exposed control samples) and quantify the biological impact of exposure by combining alterations in gene expression into differential network-node values, i.e., one value for each node of a causal network model [43]. Relevant network models used for the analysis in this study are listed in Supplementary Table 2. The selection of the network models was based on the relevancy to respiratory epithelium biology.

The NPA method uses transcriptome data without a fold-change or *p* value cut-off. The differential node values were determined by fitting procedures inferring the values that best satisfy the directionality of the causal relationships contained in the network model (e.g., positive or negative signs). NPA scores carried a confidence interval accounting for experimental variation, and the associated *p* values were computed. In addition, companion statistics were derived to permute the network structure, and the gene expression profiles were derived to inform the specificity of the NPA score to the biology described in the network models. The results from those permutation statistics were reported as *O and K* if their *p* values fell below the threshold of significance (0.05). A network was considered significantly affected by exposure if the three values (the *p* value for experimental variation, *O, and K*) were below 0.05 [42]. Network subgraphs (termed “NPA modules”), which are rich in leading nodes (i.e., nodes contributing the most to the NPA score of a network), were extracted by finding a maximum score of the connecting subgraph, using the sum of the leading node contributions as scores.

A system-wide metric for biological impact, the biological impact factor (BIF) [44, 45] summarized the impacts of the exposure on the cellular system into a single (absolute) number, thus enabling a simple and high-level evaluation of the treatment effects across multiple time points. Calculating the BIF required the collection of all applicable hierarchically structured network models (Supplementary Table 2), and involved aggregating the NPA score of the individual networks.

## Results

### Particle sizes of cigarette smoke and test mix, base, or carrier aerosols were similar

To compare the in vitro biological effects of the mixtures fairly, basic physical characteristics of aerosols will demonstrate that the observed effects were not influenced significantly by the particle size (the difference in particle size may bias the true effects of the exposure). Table 5 shows that the particle size measurements of the 3R4F CS, detected before entering the exposure system, were similar to those of test mix, base, and carrier aerosols.

**Table 5:** Particle size characterization of smoke or aerosols before entering the exposure system

Item	Median (µm)	Mean (µm)	Geometric mean (µm)	Mode (µm)	Geometric standard deviation
3R4F CS	2.55 ± 0.25	3.36 ± 0.26	2.72 ± 0.19	2.28 ± 0.91	1.89 ± 0.06
Test mix	2.43 ± 0.15	3.07 ± 0.18	2.59 ± 0.13	2.21 ± 0.30	1.75 ± 0.05
Base	2.49 ± 0.16	3.09 ± 0.18	2.64 ± 0.13	2.33 ± 0.26	1.72 ± 0.06
Carrier	2.42 ± 0.15	3.02 ± 0.18	2.58 ± 0.13	2.24 ± 0.24	1.71 ± 0.05

Values are reported ± standard deviation (*N* = 3–4 independent measurements)

*CS* cigarette smoke

### Concentrations of deposited carbonyls in the exposure chamber following exposure to cigarette smoke were greater than concentrations following exposure to test mix, base, or carrier aerosols

**Fig. 3:**
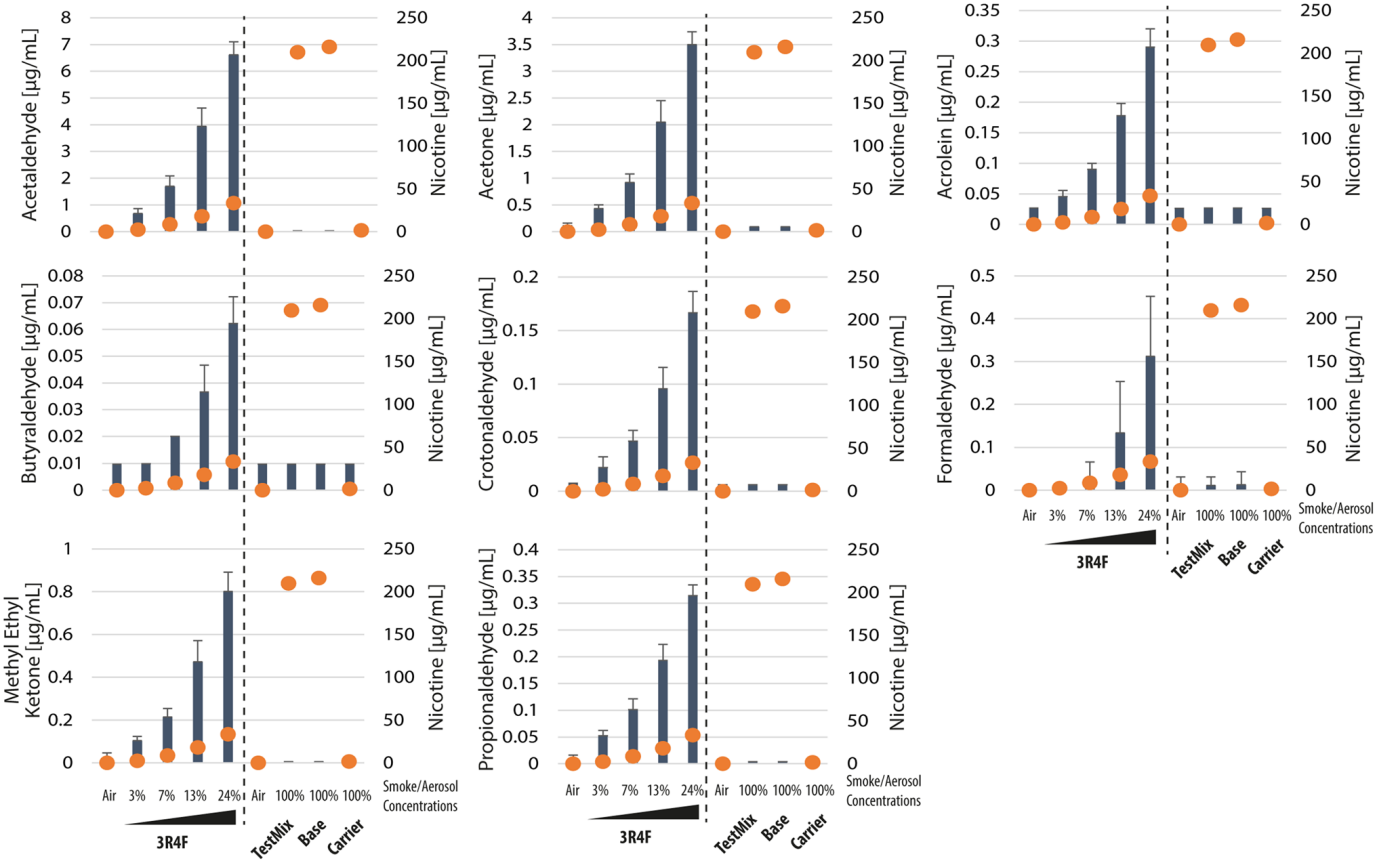
Concentrations of deposited carbonyls and nicotine in the exposure chamber. Shown are the mean and standard deviation of concentrations of deposited carbonyl compounds (left *y*-axis) after a 112-puff exposure to 3R4F cigarette smoke or test mix, base, or carrier aerosols at the indicated concentrations (*x*-axis). The orange dots indicate the median concentrations of nicotine deposited in the exposure chamber (right *y*-axis; given in Table 4)

We measured the concentrations of deposited carbonyls in the exposure chamber following a 112-puff exposure to various concentrations (from 3 to 24%) of 3R4F CS; and following a 112-puff exposure to undiluted aerosols (100%) of test mix, base, and carrier. Figure 3 shows that the concentrations of deposited acetaldehyde, acetone, acrolein, butyraldehyde, crotonaldehyde, formaldehyde, methyl ethyl ketone, and propionaldehyde following the exposure to diluted 3R4F CS were much greater than those following exposure to undiluted test mix, base, or carrier aerosols, for the same puff number. It should be noted that the concentrations of deposited nicotine following exposure to the undiluted test mix or base aerosols were 6–100 times greater than concentrations following exposure to 3–24% 3R4F CS (orange dots, Fig. 3).

### Exposure to cigarette smoke caused tissue damage, but exposure to test mix, base, or carrier aerosols did not

**Fig. 4:**
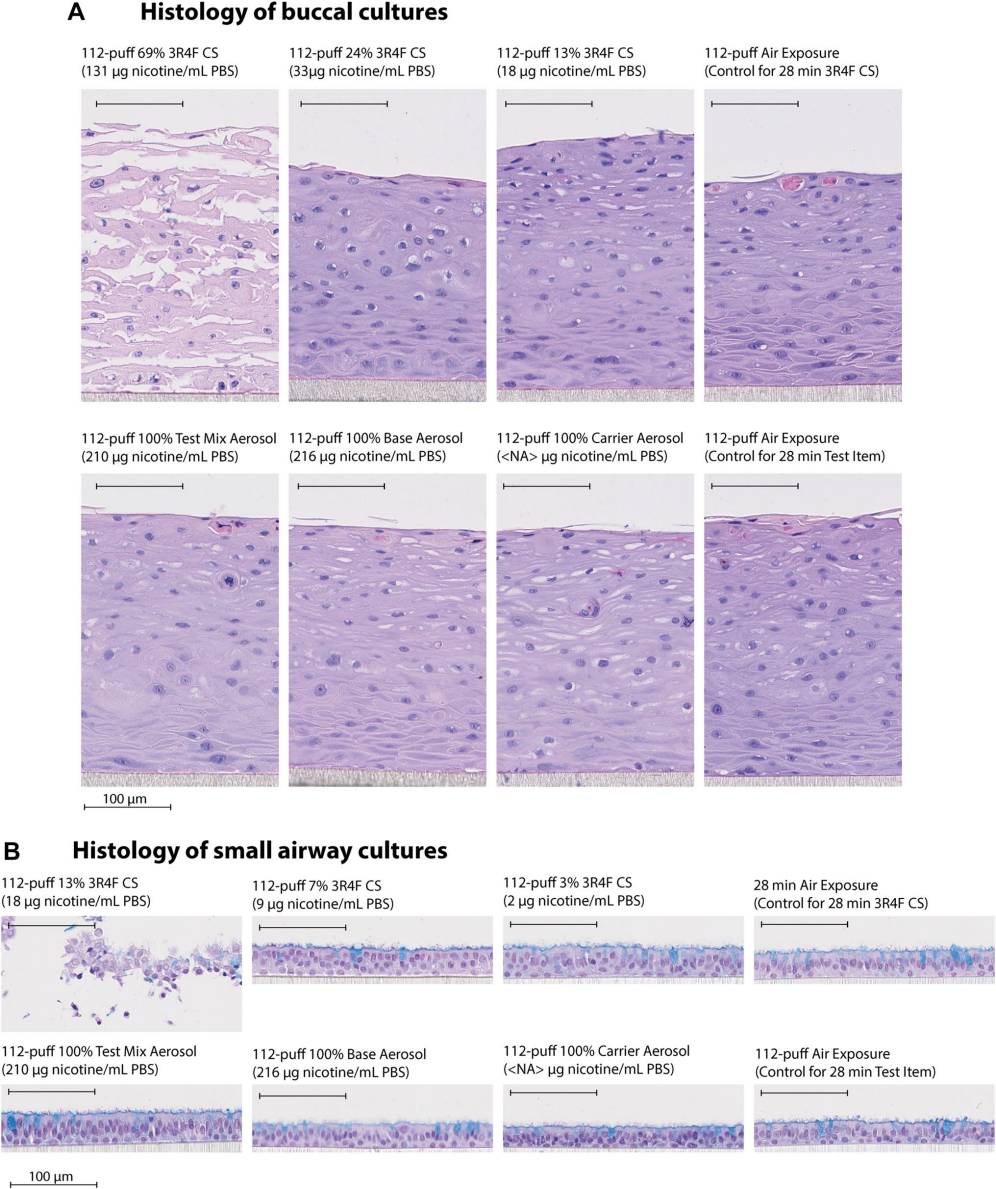
Histological characteristics of buccal and small airway cultures. Representative images of buccal (**a**) and small airway (**b**) culture sections stained with hematoxylin and eosin 48 h after exposure to diluted 3R4F cigarette smoke or undiluted test mix, base, or carrier aerosols at the indicated concentrations for 28 min (112 puffs). Small airway cultures were also stained with Alcian blue to visualize goblet cells. Bar = 100 µm. *PBS* phosphate-buffered saline

**Fig. 5:**
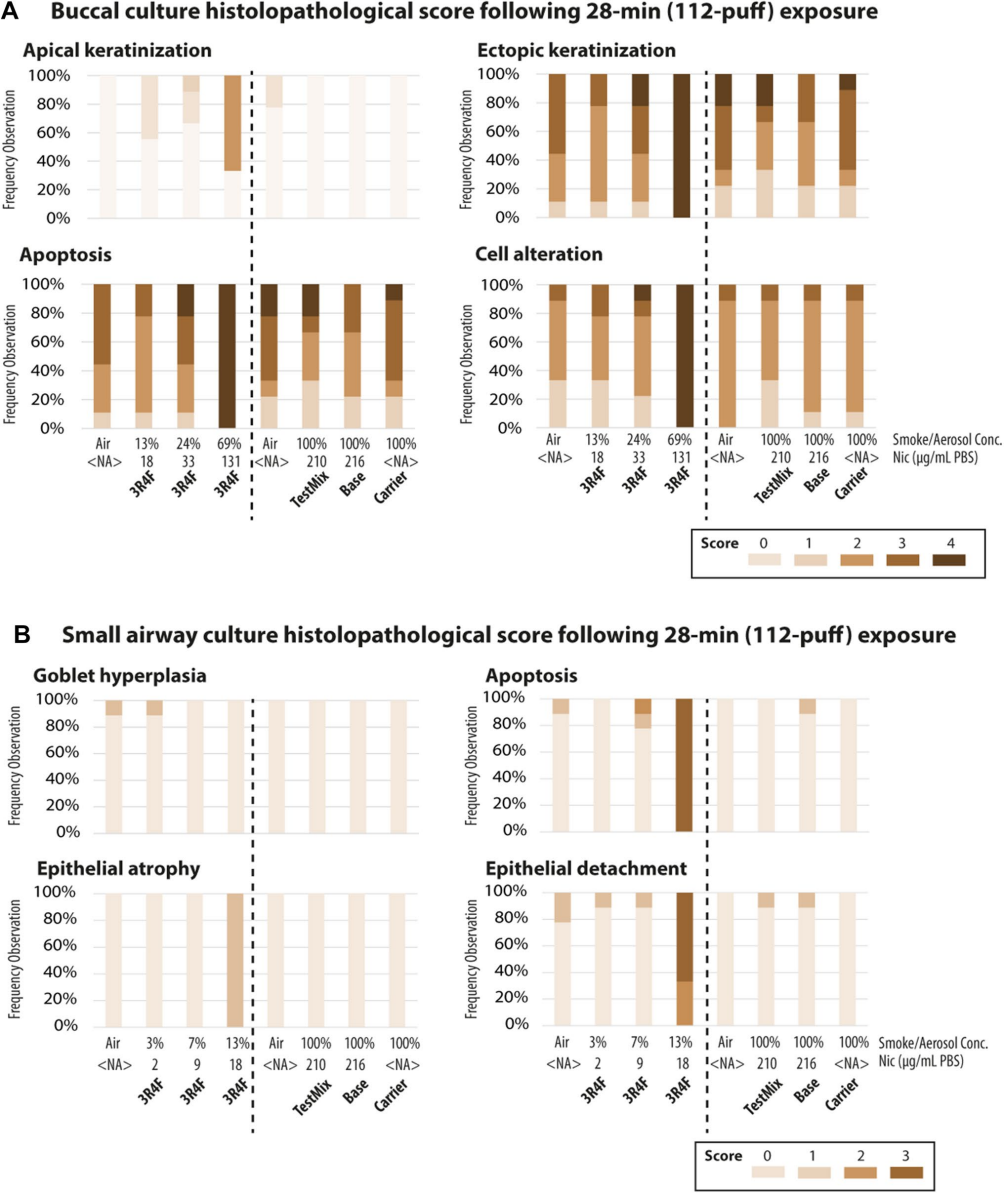
Quantification of histopathological findings. The percent distribution of the histopathological scores per treatment group is shown for buccal (**a**) and small airway (**b**) cultures. The color gradient highlights the scores of the histological findings (N = 9 independent samples, from nine independent exposure experiments). Other findings that were scored are given in Supplementary Fig. 1. The specification of the various histopathological findings that were assessed is given in Supplementary Table 1. *NA* not applicable

We determined the effects of exposure on the morphology of buccal and small airway cultures 48 h after exposure. Figure 4a shows that the 112-puff exposure to 69% 3R4F CS (resulting in a deposited nicotine concentration of 131 µg/mL PBS) caused apparent tissue damage in buccal cultures. This dose of CS resulted in the highest scores for apoptosis, ectopic keratinization, apical keratinization, and cell alteration [Fig. 5a, other findings were scored; however, marked impacts of exposure were not detected (see Supplementary Fig. 1)]. The 112-puff exposure to 3R4F CS at lower concentrations (13% and 24%) did not cause obvious damage in buccal cultures (Fig. 4a). The 112-puff exposure to 100% aerosol of test mix or base (resulting in a deposited nicotine concentration of around 200 µg/mL PBS) did not alter the culture morphology, which was similar to that of air-exposed cultures. Exposure to the aerosol of the humectants only (i.e., the carrier group did not contain nicotine or flavor ingredients) for 112 puffs did not elicit visible morphological changes.

Figure 4b shows that the 112-puff exposure to 13% 3R4F CS (resulting in a deposited nicotine concentration of 18 µg/mL PBS) caused tissue damage in small airway cultures. This dose of CS resulted in the highest score for apoptosis, epithelial detachment (from the membrane), and epithelial atrophy [Fig. 5b, other findings were scored; however, the impacts of exposure were not detected (see Supplementary Fig. 1)]. The 112-puff exposure to the lower concentrations of 3R4F CS (3 and 7%) or to the undiluted test mix or base aerosol (which resulted in a deposited nicotine concentration of approximately 200 µg/mL PBS) did not cause evident morphological alterations. Culture morphology following the 112-puff exposure to any of EC aerosols was similar to that of air-exposed cultures.

Altogether, the results showed that test mix or base aerosol exposure did not induce morphological alterations in either culture types, unlike 3R4F CS exposure, despite greater deposited nicotine concentrations at the tested doses. The effects of the carrier aerosols on culture morphology were not particularly different from the effects of the EC aerosol containing nicotine (base) or the EC aerosol containing both nicotine and flavor ingredients (test mix).

### Exposure to cigarette smoke reduced the frequency of cilia beat in small airway cultures but exposure to test mix, base, or carrier aerosols did not

**Fig. 6:**
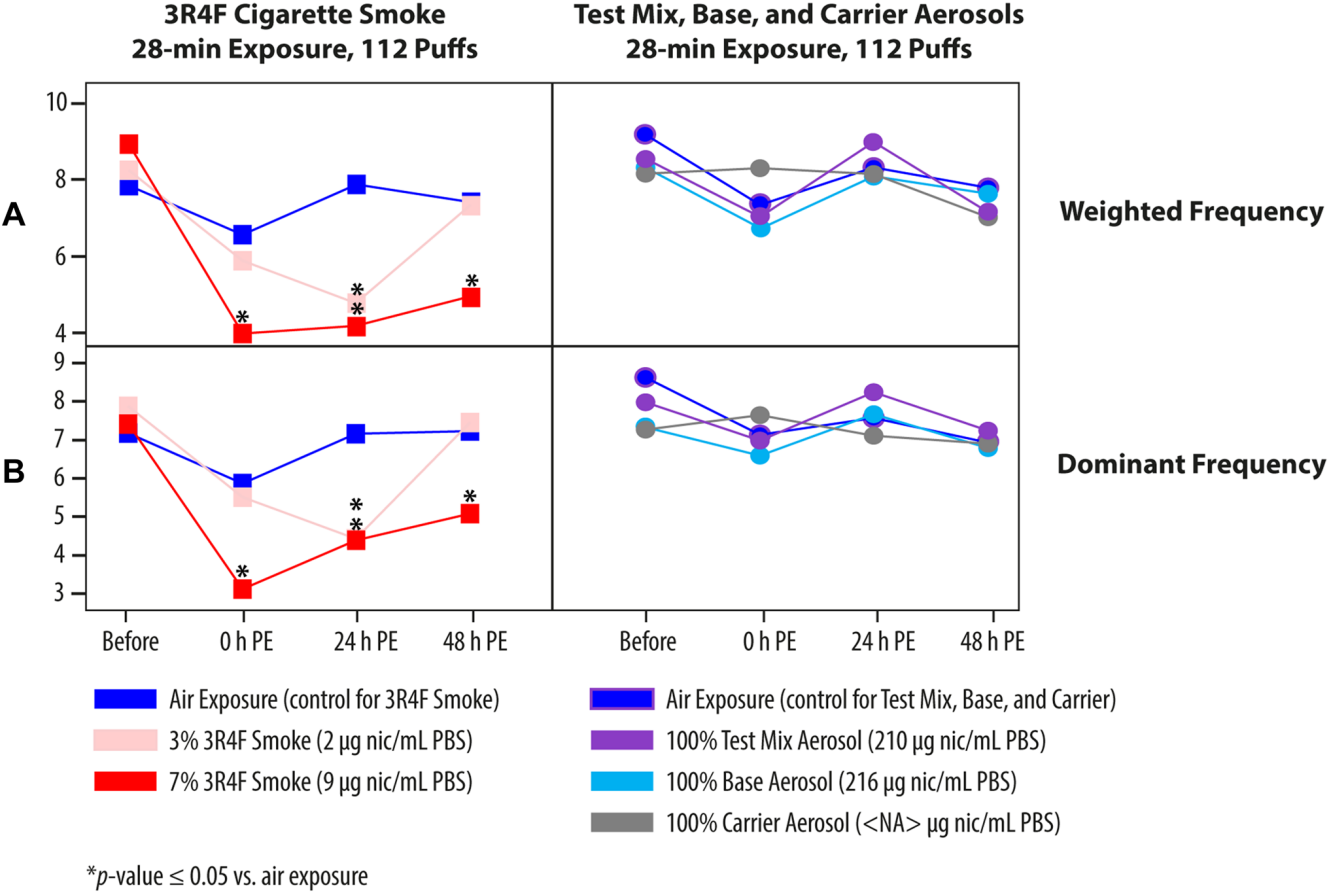
Impact of exposure on ciliary beating functionality. Ciliary beating frequency was assessed longitudinally before exposure, immediately after exposure (0 h), and 24 h and 48 h post-exposure to the diluted 3R4F CS or undiluted test mix, base, or carrier aerosols for 28 min (112 puffs). **a** Weighted frequency (Hz); **b** dominant frequency (Hz). *NA* not applicable; *PBS* phosphate-buffered saline; *PE* post-exposure; *nic* nicotine

In humans, CS exposure is known to shorten cilia length and reduce CBF [46]. To evaluate the change in CBF, we measured the frequency before exposure, immediately after (0 h post-exposure), and 24 h and 48 h post-exposure to the diluted 3R4F CS or undiluted test mix, base, or carrier aerosols. Figure 6 shows that 3R4F CS exposure resulted in a dose-dependent decrease in cilia beat frequency. The cultures exposed to the 13% 3R4F CS were not analyzed because of the obvious overt tissue damage (see Fig. 4b). Immediately following exposure, a dramatic drop in CBF was detected, particularly following exposure to the 7% 3R4F CS (Fig. 6). In contrast, CBF was not significantly altered following exposure to the undiluted test mix, base, or carrier EC aerosols.

### Mechanistic investigation of the biological effects of the exposure to 3R4F cigarette smoke and test mix, base, and carrier aerosols

The aforementioned results showed that the 112-puff acute exposure to diluted 3R4F CS (69% to buccal cultures and 13% to small airway cultures) caused tissue damage, while 112-puff exposure to test mix, base, or carrier aerosols (100%, undiluted) did not. We considered that these 3R4F CS doses were not suitable for mechanistic investigation, because cellular and molecular changes in damaged cultures would simply reflect the already perceived pronounced morphological changes [47, 48]. Accordingly, only cultures exposed to the subtoxic doses of 3R4F CS were used for the mechanistic investigation. For buccal cultures, the subtoxic doses of 3R4F CS were those resulting in deposited nicotine concentrations of 18 and 33 µg/mL PBS (13 and 24% CS). For small airway cultures, the subtoxic doses were those resulting in deposited nicotine concentrations of approximately 2 and 9 µg/mL PBS (3 and 7% CS, respectively) (Fig. 4). The 112-puff exposure to test mix or base aerosols, which resulted in deposited nicotine of approximately 200 µg/mL PBS, and to carrier aerosol were considered subtoxic doses because tissue damage was not observed. Here, we present results from two different approaches to study the mechanisms of biological effect following exposure: a causal network enrichment analysis and the profiling of secreted inflammatory mediators.

**Fig. 7:**
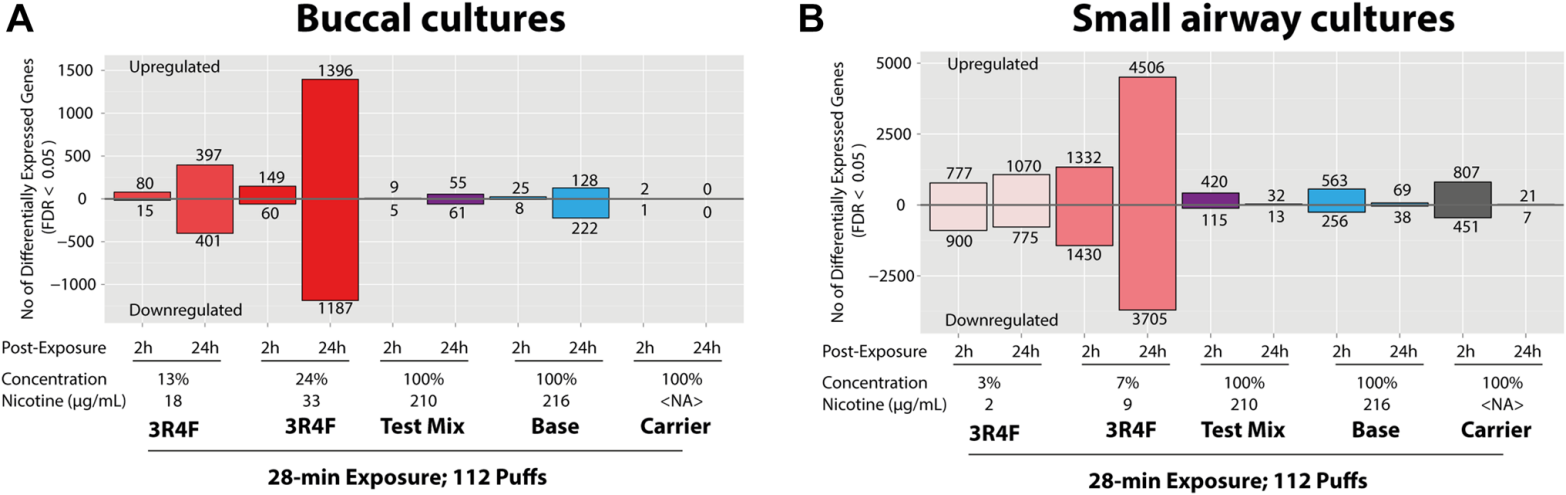
Numbers of differentially expressed genes. Each bar displays the numbers of genes significantly altered in buccal (**a**) and small airway (**b**) cultures. *y*-axis > 0 indicates increased expression and *y*-axis < 0 indicates decreased expression relative to air-exposed controls, for each exposure condition (*x*-axis). *FDR* false discovery rate; *NA* not applicable

For the first approach, using whole-genome array, we compared the expression of genes in the cultures 2 h and 24 h following exposure to subtoxic doses of 3R4F CS or to test mix, base, or carrier aerosols, with the expression of genes in air-exposed cultures. The overall expression profiles following exposure to the subtoxic doses of 3R4F CS were strikingly different from the profiles seen following exposure to aerosols of test mix, base, or carrier (Fig. 7). In both culture types, exposure to the subtoxic doses of 3R4F CS elicited the highest number of genes differentially expressed 24 h post-exposure, with a maximum of 2583 genes in buccal cultures and 8211 genes in small airway cultures.

In buccal cultures, test mix and base aerosol exposure resulted in greater numbers of genes differentially expressed than carrier aerosol exposure, although the numbers were substantially lower than those found in buccal cultures exposed to 3R4F CS, at a given post-exposure time point (Fig. 7a). In small airway cultures, the pattern of the gene expression profiles following exposure to 3R4F CS was similar to the pattern in buccal cultures, in which more genes were altered at the 24 h than at the 2 h post-exposure time point. However, following exposure to Test mix, base, or carrier aerosols, more genes were differentially expressed 2 h post-exposure than 24 h post-exposure (Fig. 7b). We also detected more genes differentially expressed following exposure to carrier aerosol than following exposure to test mix or base aerosols, albeit at much lower numbers compared with the genes altered following 3R4F CS exposure.

To examine to what extent the exposure-induced gene expression changes impacted cellular processes and/or pathways, we used a collection of causal biological network models [43]. We performed a network enrichment analysis using transcriptome data as an input, and the network perturbation amplitude (NPA) algorithm [42], a *p* value threshold-free approach, to compute exposure-induced perturbation scores of various processes modeled in these causal models. These network models were built considering defined context boundaries relevant to the biology attributable to smoking (i.e., diseased and nondiseased pulmonary and vascular tissues). Because this contextual information is typically not available from most existing knowledge bases [49], we considered that the use of these causal network models improved the specificity and sensitivity to infer subtle exposure impacts.

**Fig. 8:**
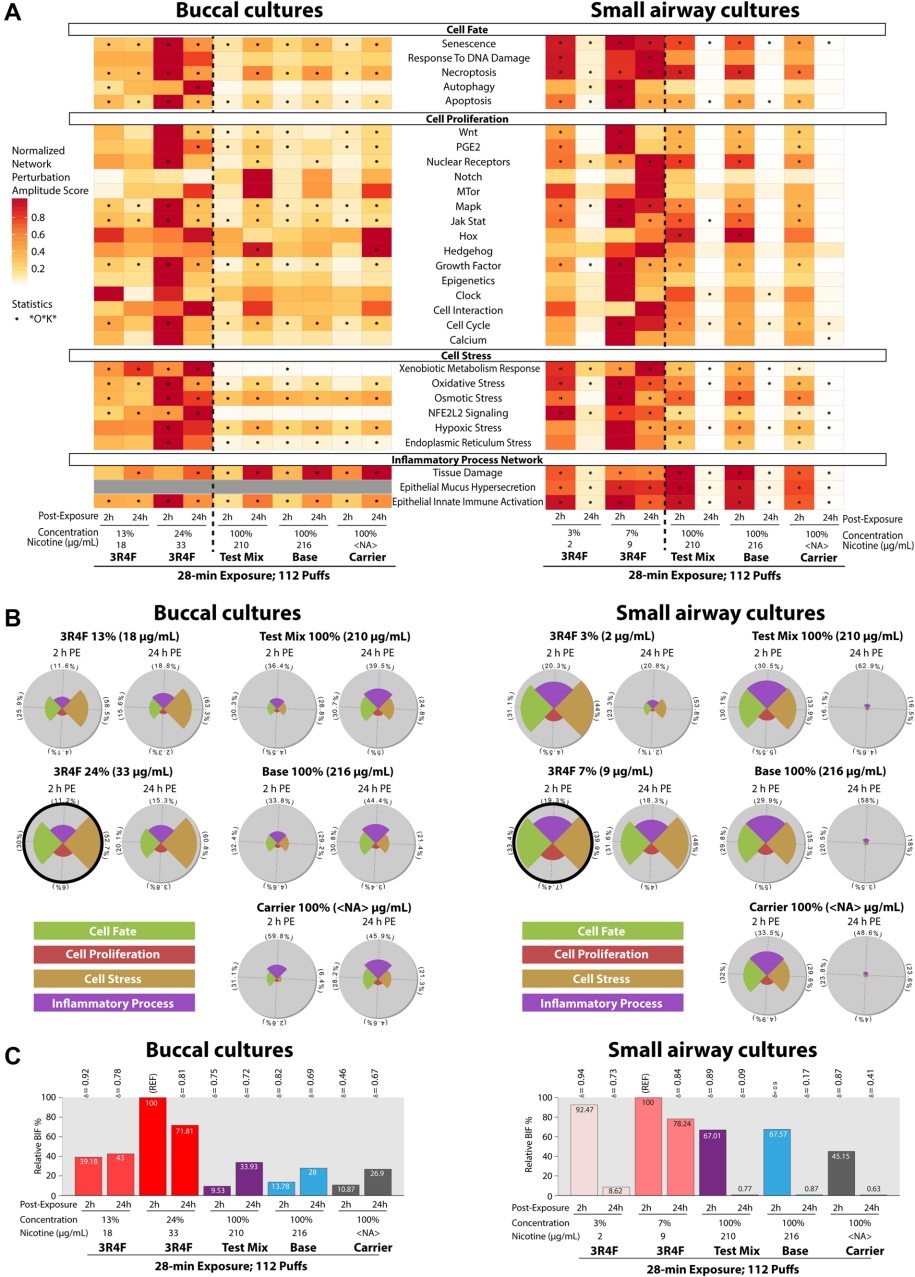
Causal network enrichment approach for the analysis of transcriptome data. **a** Perturbation scores for each network and treatment group. **b** Star plots showing the relative biological impact factor of each network family. Star plots circled in thick black lines had the highest impact factor. The slice of the plot, which is also marked in percentages, refers to its relative contribution to the overall biological impact factor (BIF, shown in **c**). **c** The relative BIF scores were normalized to the maximum impact score (marked “REF”). *PE* post-exposure; *NA* not applicable

Figure 8a shows the perturbation scores of various pathways/processes in cultures following exposure. The *Epithelial Mucus Hypersecretion* network model was not used for analyzing the buccal culture dataset because mucus is not present in buccal cultures (nor in the in vivo tissue counterpart). Figure 8a displays greater perturbation scores for the majority of the networks at the earlier (2 h) than the later (24 h) post-exposure time point in both cultures following subtoxic doses of 3R4F CS exposure. We note, however, that the pattern of perturbation scores following exposure to test mix, base, or carrier aerosols in buccal cultures was different from the pattern in small airway cultures; greater perturbation scores for most networks were detected 24 h post-exposure in buccal cultures, but 2 h post-exposure in small airway cultures. We concluded that the tissue responses following exposure to EC aerosols were tissue type dependent. Relative to the perturbation scores following exposure to the higher concentrations of 3R4F CS, the scores following exposure to the test mix, base, or carrier aerosols were substantially lower in both culture types. The analysis also showed that the test mix-induced perturbation scores were not markedly different from the base-or carrier-induced perturbation scores.

Furthermore, we summarized the exposure-induced impacts into four main cellular processes: the cell fate, cell proliferation, cell stress, and inflammatory process network families (Fig. 8b). The networks shown in Fig. 8a were assembled into these four families, and the results in Fig. 8b show the relative biological impact score for each network family. Exposure to subtoxic doses of 3R4F CS elicited greatest impact on the cell stress family, followed by cell fate, among the other families in both cultures. The impacts were also greater 2 h than 24 h post-exposure, suggesting recovery.

In buccal cultures, the impact of exposure to test mix, base, or carrier aerosols on the four network families were greater 24 h than 2 h post-exposure (Fig. 8B). The exposures affected predominantly the inflammatory process network family. Differently, in small airway cultures, we detected that cell fate, cell stress, and inflammatory process network families were similarly impacted following exposure to test mix, base, or carrier aerosols, while the cell proliferation network family was the least affected. The impact of the aerosols on all four network families disappeared almost completely at 24 h post-exposure while it persisted following exposure to 7% 3R4F CS.

We further questioned the comparability of the overall impact of exposure to undiluted test mix, base, or carrier aerosols and that of exposure to subtoxic doses of 3R4F CS. To answer this question, we computed the overall impact score (termed Biological Impact Factor, BIF) by aggregating the perturbation scores of all networks. It should be noted that the 112-puff exposure to test mix or base aerosols resulted in the deposited nicotine concentrations much greater than the concentrations following the 112-puff 3R4F CS at the tested doses. Figure 8c shows that the overall biological impacts of 3R4F CS exposure, at a given post-exposure time point, were still higher than those of exposure to the test mix, base, or carrier aerosols in both culture types. Furthermore, the overall impact score of exposure to test mix aerosol was similar to those of base or carrier aerosols.

**Fig. 9:**
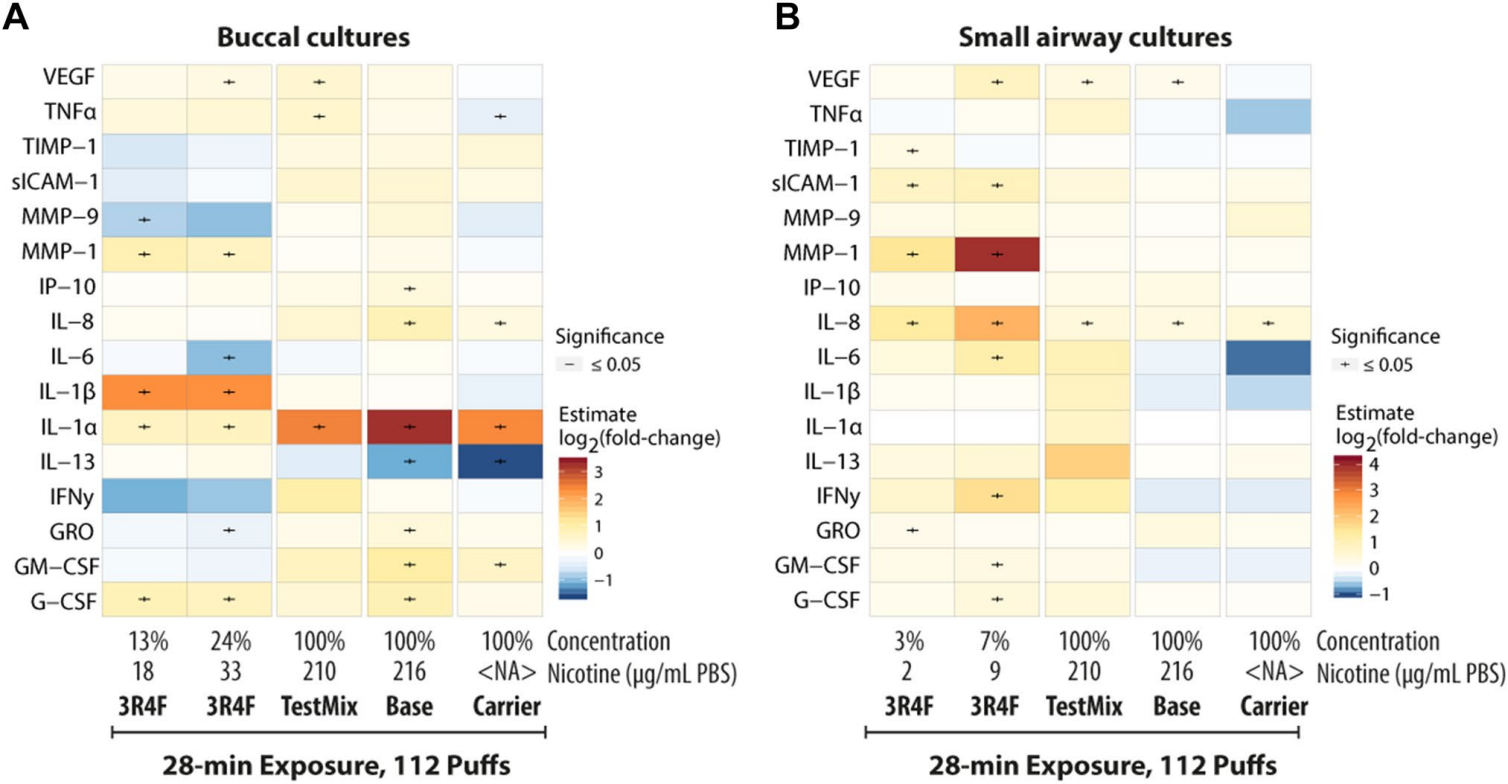
Changes in inflammatory mediator concentrations in the culture medium following exposure. Heat map color indicates the log_2_(fold-change) of the concentrations of mediators in the medium of buccal (**a**) and small airway (**b**) cultures exposed to 3R4F cigarette smoke or test mix, base, or carrier aerosols over concentrations in air-exposed samples. *NA* not applicable; *PBS* phosphate-buffered saline. Boxplot representations of the buccal and small airway data are reported in Supplementary Fig 2 and Supplementary Fig 3, respectively

We next investigated the inflammatory responses of the cultures following exposure to 3R4F CS and to test mix, base, or carrier aerosols by profiling the protein expression of secreted inflammatory mediators in the media collected 48 h post-exposure. Figure 9a, b illustrates that the overall changes in inflammatory mediators were tissue type-specific.

## Discussion

The study found significantly lower toxicity (tissue damage and CBF changes) following exposure to undiluted EC aerosols (test mix, base, or carrier) in comparison to diluted 3R4F CS, at a matching puff number, in human 3D organotypic buccal and small airway epithelial cultures. In buccal cultures, tissue damage was not seen following exposure to test mix or base aerosols despite resulting in a concentration of deposited nicotine nearly double that of 3R4F CS (Fig. 4). In small airway cultures, tissue damage and altered CBF were not apparent following exposure to test mix or base aerosols even at a deposited nicotine concentration 10–20 times that of 3R4F CS (Figs. 4, 6). The minimal impact of EC aerosol exposure on ciliary beating was consistent with a previous observation in excised bullfrog palates [46]. Lower toxicity of exposure to whole EC aerosols than to whole CS has been consistently reported by other groups [10–12, 14].

Compared with the results from studies in which only the EL or fractions of EC were tested [15–22], the results of the present study can better extrapolate the potential lower acute toxicity of EC use relative to the toxicity associated with CS use, because we tested the impact of the whole aerosol in a manner mimicking realistic inhalation exposure. It should be again noted that although undiluted EC aerosols were tested in this present study, diluted 3R4F CS was applied to the cultures to avoid overt tissue damage, thus allowing mechanistic investigation.

The study included nine independent aerosol generations (for each culture type); the phase repetitions were performed to allow for drawing robust conclusions, despite using data from only one donor. We obtained a good correlation of the fold-changes in the gene expression across the repetition phases (Supplementary Fig. 4). We also had previously reported that the pattern of gene expression changes was comparable between cultures from two donors following CS exposure [35]. Furthermore, Banerjee et al. reported that a similar trend of inflammatory mediator secretion was detected in cultures from six different donors following CS exposure [50]. These studies thus support that the results of the present study, despite using data from one donor, can still provide valuable insights into the underlying mechanisms associated with CS and EC aerosol exposures.

### Dose selection

Despite the lack of a standardized protocol for the generation of an EC aerosol, we adapted a similar parameter to the one used for the generation of CS. The doses of 3R4F CS used in the present study were selected based on our previous studies, in which organotypic cultures were exposed to 112 puffs (28 min) of CS. These doses have been shown consistently to be subtoxic and toxic to buccal cultures (at ≤ 24% and 69% 3R4F CS, respectively) [33, 34] and small airway cultures (at ≤7% and 13% 3R4F CS, respectively) [31, 32]. Because EC aerosol generally “contains fewer numbers and lower levels of most toxicants than smoke” [3], we opted not to dilute the aerosol, i.e., 100% EC aerosols were administered to cells despite knowing that the EC aerosol exposure would result in greater deposited nicotine concentrations. When compared to the reported EC usages in literature, the number of puff tested in the present study (112 puffs) was comparable to the median puff number (132 puffs/day) measured in a group of 135 French EC users, with a mean puff duration of nearly 4 s [51]. This mean puff duration is comparable to the duration used in the present study (5 s), which was used also in a previous in vivo study [52].

The concentrations of deposited nicotine in the chamber following exposure were used to offer a perspective on the amount of nicotine delivered, which is relevant in the context of tobacco harm-reduction strategy. The use of other sources of nicotine besides CS should reduce the exposure to harmful constituents by eliminating the inhalation of the toxic compounds generated when tobacco is burned at high temperatures, as it is in CS [53]. Our data showed that, although exposure to test mix and base aerosols resulted in greater concentrations of deposited nicotine, the concentrations of carbonyls deposited in the exposure chamber following exposure to the EC aerosols were substantially lower than those deposited following exposure to 3R4F CS at the tested doses. Studies have reported that the generation of carbonyls, from an EC emission, is influenced by the device. For example, new atomizers with better wicking material resulted in lower carbonyl emissions, even lower than the environmental levels and occupational safety limits [54]. Furthermore, in the present study, we measured the compound deposition in PBS samples. These PBS samples were placed in the exposure chamber together with the cultures during an exposure experiment. We acknowledge that the deposition in PBS does not reflect the compound deposition on to ALI cultures; however, it can be regarded as a proxy for the quantities deposited onto the cultures.

Compared with the concentrations of nicotine found in the saliva of EC users (reaching a maximum of 0.86 µg/mL) [55], the concentrations of nicotine deposited following exposure to the test mix or base EC aerosols in the present in vitro study were about 200 times higher (we detected approximately 200 µg nicotine/mL PBS following the 112-puff exposure to test mix or base aerosols). We recognize that the deposited doses in vitro (in the exposure chamber) cannot be directly translated to the in vivo situation (in the human buccal or small airway epithelia) because local dosimetry [e.g., air flow, apical liquid (saliva/mucus) flow] influences the actual deposition of compounds in the human tissues. Better extrapolation of the in vitro–in vivo dosimetry could be achieved in the future by performing complementary work using computational modeling approaches, for example, using whole-lung airway models [56–59].

### Tissue-specific molecular alterations

Distinct cellular response patterns were evident between the buccal and small airway cultures following exposure. These tissue-specific responses were not apparent from the observed alterations to the culture morphology; exposure to the test mix, base, or carrier EC aerosols did not lead to tissue damage in either buccal or small airway cultures. However, based on the transcriptome data, we detected that test mix, base, and carrier EC aerosols impacted predominantly inflammatory response in buccal cultures (reflected by the increased perturbation scores of the inflammatory process network family) that persisted until 24 h post-exposure. In small airway cultures, the cell fate, cell stress, and inflammatory process network families were equally impacted; however, they were mainly detected at the 2 h post-exposure time point and recovered almost completely 24 h post-exposure (i.e., the impact scores were extremely low). The pattern of the overall impact—the BIF scores derived from the transcriptome profiles—following exposure to the EC aerosols suggested that small airway cultures could be more sensitive in responding to insults but recovered more rapidly than buccal cultures. The more rapid recovery in small airway cultures than in buccal cultures following exposure, may be attributed to the presence of mucus layer on the apical side of small airway cultures. Furthermore, the delayed response (recovery) in buccal cultures may be attributed to the thicker and squamous structure of the epithelium. This tissue-specific response is consistent with the notion that the structural and functional properties of an epithelium depend on its location [60]. The injury response in oral mucosa has been reported to be more rapid than in skin epithelium despite both being stratified epithelium [61]. Our observation in the present study demonstrated that tissue-specific responses can be detected in vitro. Also, the results revealed the importance of evaluating more than single time point following exposure.

Our finding that EC aerosol exposure resulted in a smaller number of differentially expressed genes (relative to gene expression following exposure to air) than exposure to CS, was consistent with findings in the previous studies that used human bronchial ALI cultures [11, 62]. We confirmed the observation of Moses et al. that exposure to nicotine-containing EC aerosols alters *CYP1A1* and *CYP1B1* gene expression; however, we detected that these changes only occurred in small airway cultures and only at the 2-h post-exposure time point, but not 24 h (Supplementary Fig. 5). Nevertheless, the network-based enrichment analysis in the present study suggested a lack of meaningful differences in the biological processes altered following exposure to test mix, base, or carrier EC aerosols.

Furthermore, the concentrations of inflammatory mediators detected in small airway culture media 48 h following exposure to test mix, base, or carrier EC aerosols were negligible; compared with the concentrations detected following air exposure, the concentrations secreted following the EC aerosols were not greatly altered. In buccal cultures, however, we detected more inflammatory mediators that were secreted into the basolateral media 48 h post-exposure to the EC aerosols, further suggesting that the response to EC aerosol exposure was culture type-specific. The secretion of distinct mediators, between those in small airway and buccal cultures, was also observed following 3R4F CS.

### In buccal cultures, EC aerosol exposure elicited inflammatory response distinct from CS exposure

While multiple studies have been performed to test the effects of EC on lung epithelial cells, the present study also evaluated the potential toxicity of exposure to EC aerosols in the human oral epithelium. With the rapid increase in EC use, the National Institute of Dental and Craniofacial Research (NIDCR) has noted that studies examining the synergistic effect of exposure to various chemical mixtures generated by EC on oral and periodontal epithelial cells are lacking [63]. Thus, our present study is aligned with the NIDCR Strategic Plan 2014–2019 and its initiatives to encourage studies assessing the effects of EC aerosols on oral health.

**Fig. 10:**
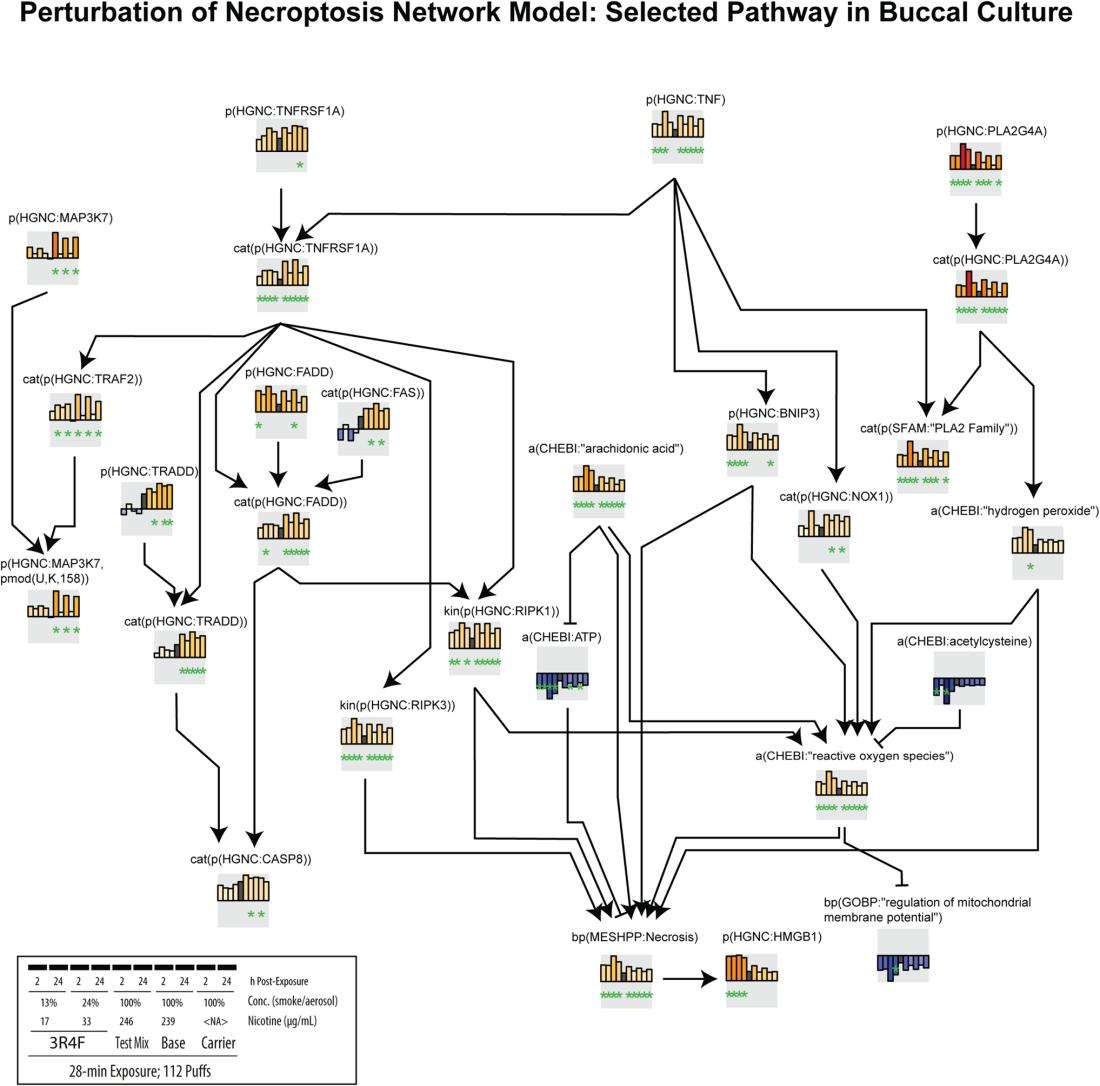
Perturbation scores of necroptosis network model in buccal cultures. The network perturbation amplitude (NPA) module (see “Materials and methods”) coded in Biological Expression Language and consisting of the nodes contributing most to the perturbation of the *Necroptosis* network in buccal cultures are shown. Color of the bars indicates node score (darker red signifies greater impact on the node and darker blue signifies lower impact on the node, relative to the impact of the air exposure). Inset shows the specification of the group pertaining to each bar plot of the node scores. A star underneath a differential node value indicates that the node was identified as a leading node

In buccal cultures, the profile of inflammatory mediators revealed that the CS- and EC aerosols-induced inflammatory responses were distinct. We detected that secretion of IL-1β increased following exposure to 3R4F CS, suggesting that the CS-induced inflammatory response could be largely mediated by inflammasomes. Inflammasome-derived IL-1β secretion can occur not only in neutrophils and macrophages, but also in epithelial cells lining the organs that are most proximal to the external environment [64]. This has been demonstrated in gingival epithelial cells following infection [65, 66]. This finding—CS-induced inflammasome activation—was not surprising because reactive oxygen species, which are produced following combustion of tobacco, are a known trigger of inflammasome activation and IL-1β secretion [67]. We consistently detected increases in IL-1β secretion following exposure to CS in our previous studies using buccal cultures [33, 34]. Furthermore, the network-based enrichment analysis indicated higher impact scores of the *p(HGNC: HMGB1)* node following 3R4F CS exposure (Fig. 10), suggesting increased levels in HMGB1 protein, which has also been linked to inflammasome activation. In retinal tissues of an acute glaucoma mouse model, HMGB1 promotes inflammasome activation and induces IL-1β processing, eliciting subsequent injury [68]. Although we cannot confirm this hypothesis in the present study, future studies should be conducted to examine the role of inflammasome in the CS-induced toxicity effects in buccal epithelia, specifically.

Differently, exposure to test mix, base, or carrier EC aerosols led to increased secretion of IL-1α in buccal cultures. Both IL-1α and IL-1β bind to the same receptor (IL-1R1) but have distinct roles. Cells that are not moribund, or exposed to nonlethal stress, have been reported to secrete IL-1α as a stress-sensing mechanism, although the release of IL-1α has been generally known to occur in necrotic cells [69, 70]. Based on the network-based enrichment analysis, exposure to the test mix, base, or carrier aerosols indeed had a considerably lower impact on necroptosis (i.e., the *bp(MSHPP:Necrosis)* node score) than exposure to 3R4F CS (Fig. 10); this was consistent with the absence of tissue damage following EC aerosol exposure (Fig. 4a). IL-1α, under sterile inflammation, is released extracellularly, leading to the production of other inflammatory mediators to recruit other inflammatory cells [71]. This concept may explain the observed increased secretions of mediators in the media of buccal cultures following exposure to test mix, base, or carrier EC aerosols, despite the lack of tissue damage. Thus, it could be speculated that the increased secretion in IL-1α levels following test mix, base, or carrier EC aerosol exposure was a part of stress-sensing mechanism in buccal cultures.

## Conclusion

Overall, the study demonstrated that exposure to undiluted test mix or base EC aerosols under the testing conditions (an acute 28-min exposure), even at a deposited nicotine concentration that is 200 times greater than that found in the saliva of EC users, had no impact on morphology of buccal and small airway cultures. In contrast, following the same puff number, the already diluted 3R4F CS resulted in overt tissue damage. We found that exposure to the EC aerosols did elicit changes at the cellular and molecular levels; test mix, base, or carrier aerosol exposure triggered alterations in gene expression and the profiles of secreted inflammatory mediators. Most changes, however, were much smaller than those observed following exposure to diluted CS. The results did not reveal meaningful differences in the overall impacts of exposure to test mix, base, or carrier EC aerosols that were deemed biologically relevant, but distinct patterns of molecular and cellular changes were evident between buccal and small airway cultures following EC aerosol exposure at the tested dose.

## Electronic supplementary material

Below is the link to the electronic supplementary material.

Supplementary file1 (DOCX 1458 kb)
