# Supplementary material for: A lower impact of an acute exposure to electronic cigarette aerosols than to cigarette smoke in human organotypic buccal and small airway cultures was demonstrated using systems toxicology assessment
Source: Intern Emerg Med. 2019 Mar 5;14(6):863–83. doi: 10.1007/s11739-019-02055-x (PMC6722047; doi:10.1007/s11739-019-02055-x)
Supplement: Supplementary file 1 — Supplementary file1 (DOCX 1458 kb) [file 11739_2019_2055_MOESM1_ESM.docx]

# Supplementary Materials

Supplementary Table 1. List of Findings for the Histopathological Assessment

| **Culture Type** | **Findings** | **Explanations** | **Grading** |
| --- | --- | --- | --- |
| Buccal | Cell alteration | Cellular alteration is synonymous to cellular / nuclear changes that may represent a reactive alteration, or dysplasia. Includes, nuclear enlargement, loss of normal stratification (nuclei becoming smaller with maturation), multinucleation, which may be associated with formation of pearls and dyskeratosis etc. | 6-grade score  0: none  1: mild  2: mild-mod  3: moderate (mod)  4: mod-severe  5: severe |
| Buccal | Apoptosis (single cell necrosis) and pyknosis (seen typically with dyskeratosis and parakeratosis) | The term single cell necrosis was included to cover single, non-viable cells that do formally not meet the criteria of apoptosis. Apoptosis: focally or multi-focally occurring single cell lesions characterized by shrunken / rounded cells with eosinophilic cytoplasm and condensed and or fragmented nuclear chromatin including the formation of apoptotic bodies | 6-grade score  (over length of tissue disc, both halves = 13 mm)  0: none  1: <20  2: 20-40  3: 40-60  4: 60-80  5: 80->100 |
| Buccal | Apical keratinization  (typically parakeratosis) | Keratinization, apically located | 4-grade score  (parakeratosis)  0: none  1: subtle  2: 1-2 cell thick  3: 3-4+cells thick  (NB: see at edge of most) |
| Buccal | Ectopic keratinization (dyskeratosis) | Keratinization within the epithelium | 6-grade score  (over length of tissue disc, both halves = 13 mm)  Typically correlates with apoptosis / pyknosis  0: none  1: <20  2: 20-40  3: 40-60  4: 60-80  5: 80->100  (often superficial) |
| Buccal | Cytoplasmic vacuolation | Cellular vacuolation | 5-grade score  0: none  1: mild  2: mild-moderate  3: moderate  4: severe |
| Buccal | Cysts  (difficult to assess because cytoplasmic clearing looks similar, record when >several cells across) | Empty round spaces, diameter approx 4-5 cells broad | 3-grade score  0: none  1: few (1-2)  2: >2  (often seen at edge in most) |
| Buccal | Increased intracellular fine granular basophilic material  (hypergranulosis) | Intracellular, basophilic granules, most likely related to cytoplasmic granules normally found in keratinized epithelium (stratum granulosum).  Affected cells not confined to dedicated layer, focal to diffuse.  (keratohyaline granules) | 5-grade score  0 – none  1 – barely discernible  2 – subtle  3 – readily identified / frequent (mild)  4 – easily identified / frequent (mod) |
| Buccal | Detachment of tissue above basal cell level  (often pathological, except at edge where it seems often artefactual) | The epithelial detachment above basal cell layer points at a cytotoxic effect. Cytotoxicity within a planar section of the epithelium is likely to impair cellular adherence. | 5-grade score  0 – none  1 – subtle split (seems almost spongiotic)  2 – focal/mild  3 – mod  4 – extensive |
| Buccal | Desquamation | Focal to diffuse finding, confined to the epithelium, not extending to the substrate. Includes increased apical desquamation characterized by loosening of the apical most epithelial layers of tissue and instances of (resulting) erosions. Exceeds the normally occurring grades of desquamation, therefore the finding was termed increased desquamation. | 3-grade score  0 – none or minimal (flakes)  1 – present, few cell layers  2 – present >2 layers |
| Buccal | Longitudinal cleft formation | Formation of longitudinal clefts within the tissue, sometimes showing keratinization. | 4-grade score  0 – none  1 – subtle / mild  2 – moderate  3 - severe |
| Small Airway | Apoptosis | The occurrence of apoptotic cells was deemed to be related to tissue damage. Apoptosis: focally or multi-focally occurring single cell lesions characterized by shrunken/ rounded cells with eosinophilic cytoplasm and condensed and or fragmented nuclear chromatin including the formation of apoptotic bodies. | 4-grade score  0: Not present  1: Mild, 1 -2 mitoses/HPF  2: Moderate, 2-4 mitoses/HPF  3: Severe, >4 mitoses/HPF  (HPF, high power field) |
| Small Airway | Crypts/cysts | Round or oval spaces within the epithelium of various sizes with no connection to the apical surface (cyst) or have contact to the apical surface (crypt). Crypts/cysts could be lined with epithelium, or be optically empty, or filled e.g. with Alcian blue-positive mucus. | 2-grade score  0: indicates absent  1: indicates present |
| Small Airway | Epithelial atrophy | The term describes the state of the tissue thickness and appearance. Generally, epithelial atrophy may be linked to trophic changes following irritants or to an attempted tissue regeneration following tissue defect. Therefore, reduced tissue thickness following incomplete tissue repair could reflect atrophic changes of the epithelium. | 4-grade score  0: Normal appearance  1: Mild  2: Moderate  3: Severe |
| Small Airway | Epithelial detachment | The epithelial detachment above basal cell layer points at a cytotoxic effect. Cytotoxicity within a planar section of the epithelium is likely to impair cellular adherence. Under the conditions of this study the finding is deemed to be related to impaired intercellular adhesion, and thus may represent an exposure related finding | 4-grade score  0: Not present  1: One site  2: multiple sites  3: entire strip detached |
|  | Goblet cell hyperplasia (proliferation) | The finding relates to the increase in cell number i.e., the Alcian blue-staining goblet cells. | 2-grade score  0: Similar levels to incubator controls  1: Increased AB staining goblet cells compared with incubator controls |
| Small Airway | Goblet cell hypertrophy (distention/halo) | The finding relates to enlarged goblet cells with altered (e.g. granular appearance) cytoplasm or altered homogeneity of the alcian blue-positive staining, with enlargement to multilocular structures that eventually exhibit a cystic structure (ciliated or non-ciliated). | 4-grade score  0: Normal appearance  1: Mild, 1-2 cells  2: Moderate: 2-4 cells  3: Severe: >4 cells |
| Small Airway | Loss of ciliated cells | The term refers to the loss of cilia (also referred to as “deciliation”) and/or to the loss of ciliated cells. | 4-grace score  0: Normal appearance:  1: Mild, <10% cells  2: Moderate, 10-50%  3: Severe, >50% cells |

Supplementary Table 2. List of network models used in the analysis

| Number | Abbreviated  network  family name | Network name | Used in buccal culture analysis | Used in small airway culture analysis |
| --- | --- | --- | --- | --- |
| 1 | CFA | Apoptosis | ✓ | ✓ |
| 2 | CFA | Autophagy | ✓ | ✓ |
| 3 | CFA | Necroptosis | ✓ | ✓ |
| 4 | CFA | Response to DNA Damage | ✓ | ✓ |
| 5 | CFA | Senescence | ✓ | ✓ |
| 6 | CPR | Calcium | ✓ | ✓ |
| 7 | CPR | Cell Cycle | ✓ | ✓ |
| 8 | CPR | Cell Interaction | ✓ | ✓ |
| 9 | CPR | Clock | ✓ | ✓ |
| 10 | CPR | Epigenetics | ✓ | ✓ |
| 11 | CPR | Growth Factor | ✓ | ✓ |
| 12 | CPR | Hedgehog | ✓ | ✓ |
| 13 | CPR | Hox | ✓ | ✓ |
| 14 | CPR | Jak Stat | ✓ | ✓ |
| 15 | CPR | MAPK | ✓ | ✓ |
| 16 | CPR | mTOR | ✓ | ✓ |
| 17 | CPR | Notch | ✓ | ✓ |
| 18 | CPR | Nuclear Receptors | ✓ | ✓ |
| 19 | CPR | PGE2 | ✓ | ✓ |
| 20 | CPR | Wnt | ✓ | ✓ |
| 21 | CST | Endoplasmic Reticulum Stress | ✓ | ✓ |
| 22 | CST | Hypoxic Stress | ✓ | ✓ |
| 23 | CST | NFE2L2 Signalling | ✓ | ✓ |
| 24 | CST | Osmotic Stress | ✓ | ✓ |
| 25 | CST | Oxidative Stress | ✓ | ✓ |
| 26 | CST | Xenobiotic Metabolism Response | ✓ | ✓ |
| 27 | IPN | Epithelial Innate Immune Activation | ✓ | ✓ |
| 28 | IPN | Epithelial Mucus Hypersecretion | <NA> | ✓ |
| 29 | IPN | Tissue Damage | ✓ | ✓ |

Abbreviations: CFA, Cell Fate; CST, Cell Stress; CPR, Cell Proliferation; IPN, Inflammatory Process Networks; Jak Stat, janus kinase/signal transducers and activators of transcription; MAPK, mitogen-activated protein kinases; mTOR, mechanistic target of rapamycin; NA, not applicable; NFE2L2, nuclear factor-erythroid 2 p45-related factor 2, NRF2; PGE2, prostaglandin E2. The collection of causal biological networks used here was the human network suite CBN v1.3 [[43](#_ENREF_43)].

Supplementary Figure 1. Quantification of histopathological findings (all findings)


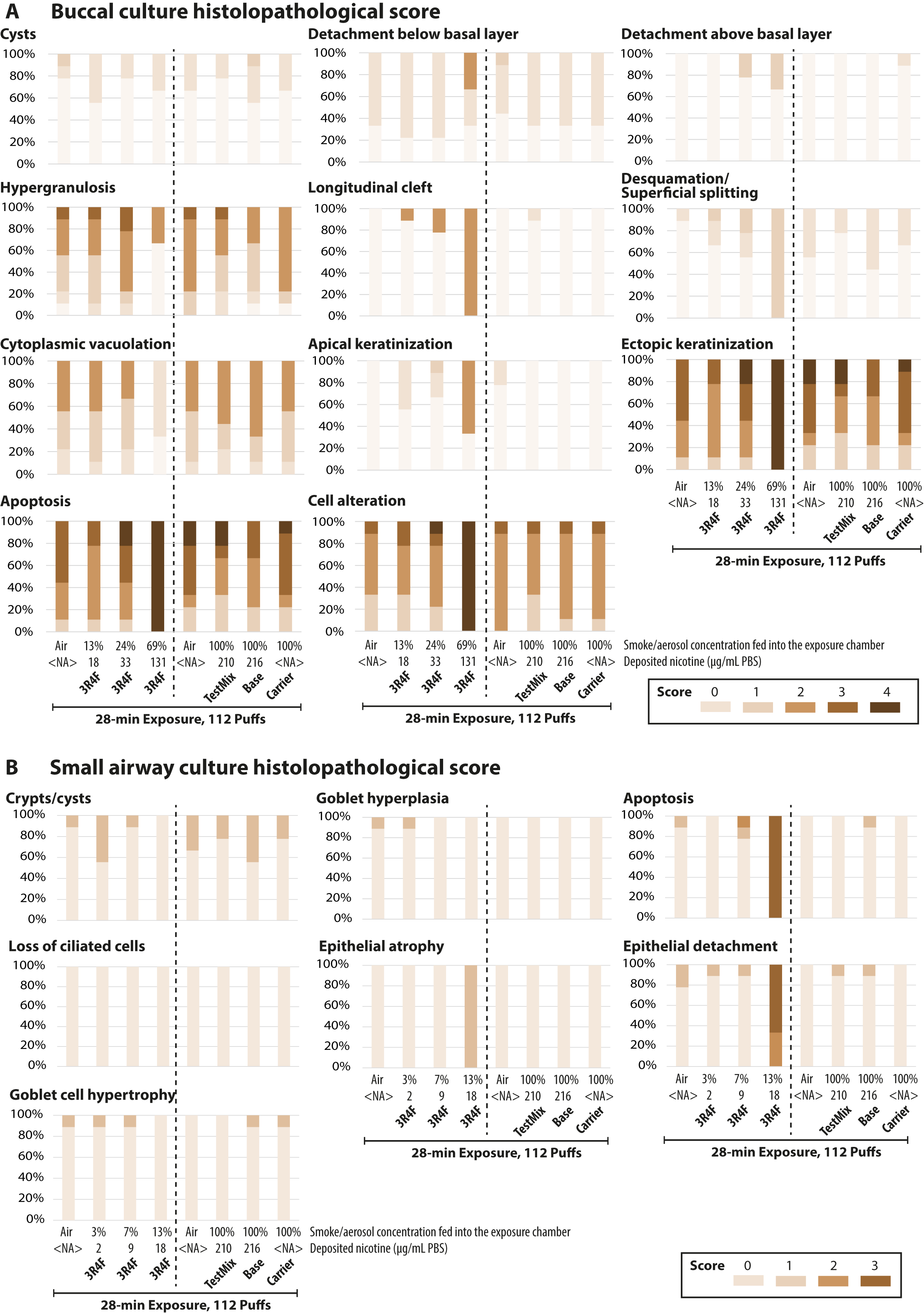


Supplementary Figure 2. Box and wisher plots of the absolute concentrations (pg/mL, y-axis) of selected mediators from buccal epithelial cultures

**p*-value ≤ 0.05 compared with the levels in the air-exposed controls. NA, not applicable; LOQ, lower limit of quantification; LOD, lower limit of detection.


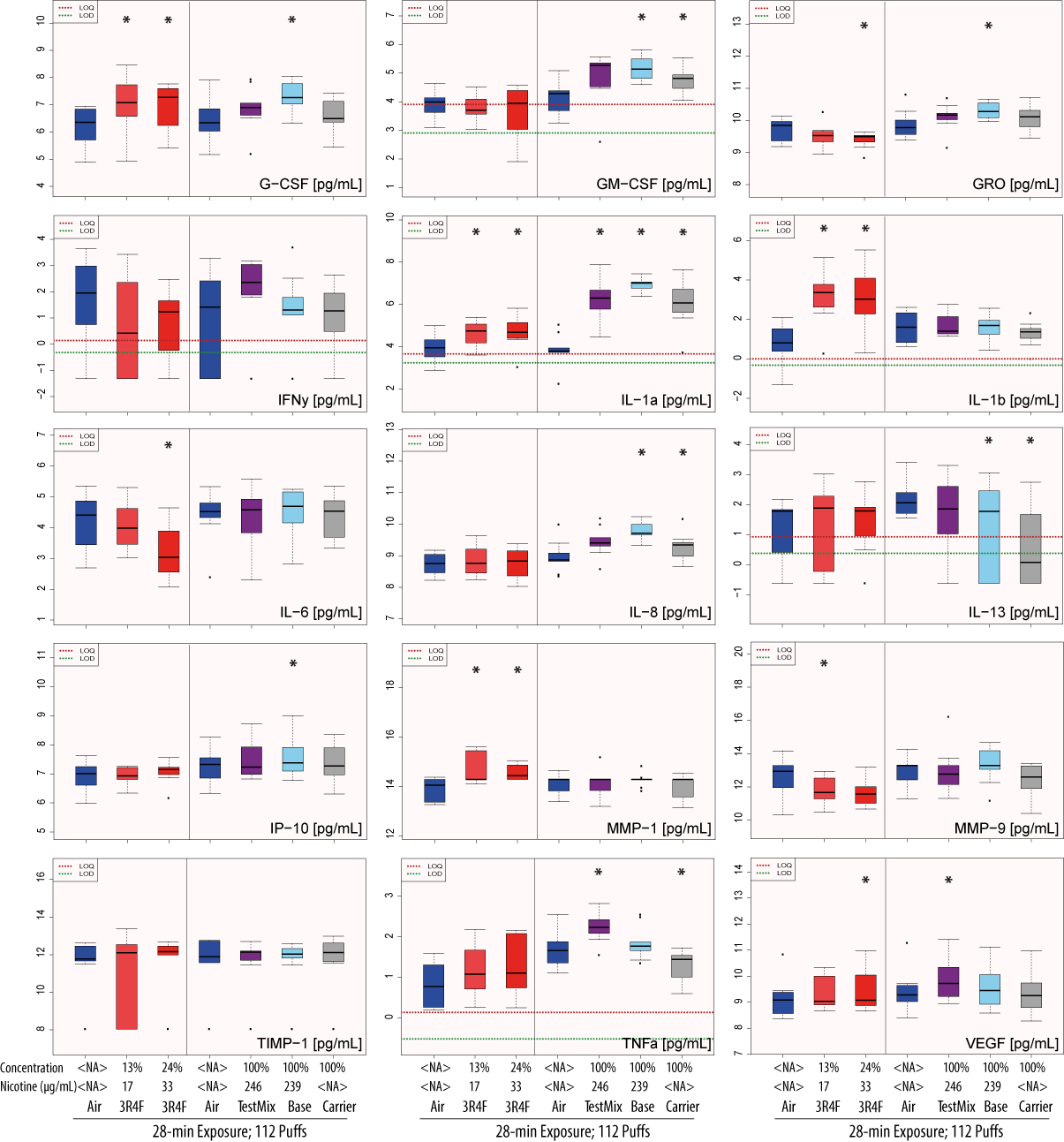


Supplementary Figure 3. Box and wisher plots of the absolute concentrations (pg/mL, y-axis) of selected mediators from small airway epithelial cultures

**p*-value ≤ 0.05 compared with the levels in the air-exposed controls. NA, not applicable; LOQ, lower limit of quantification; LOD, lower limit of detection.


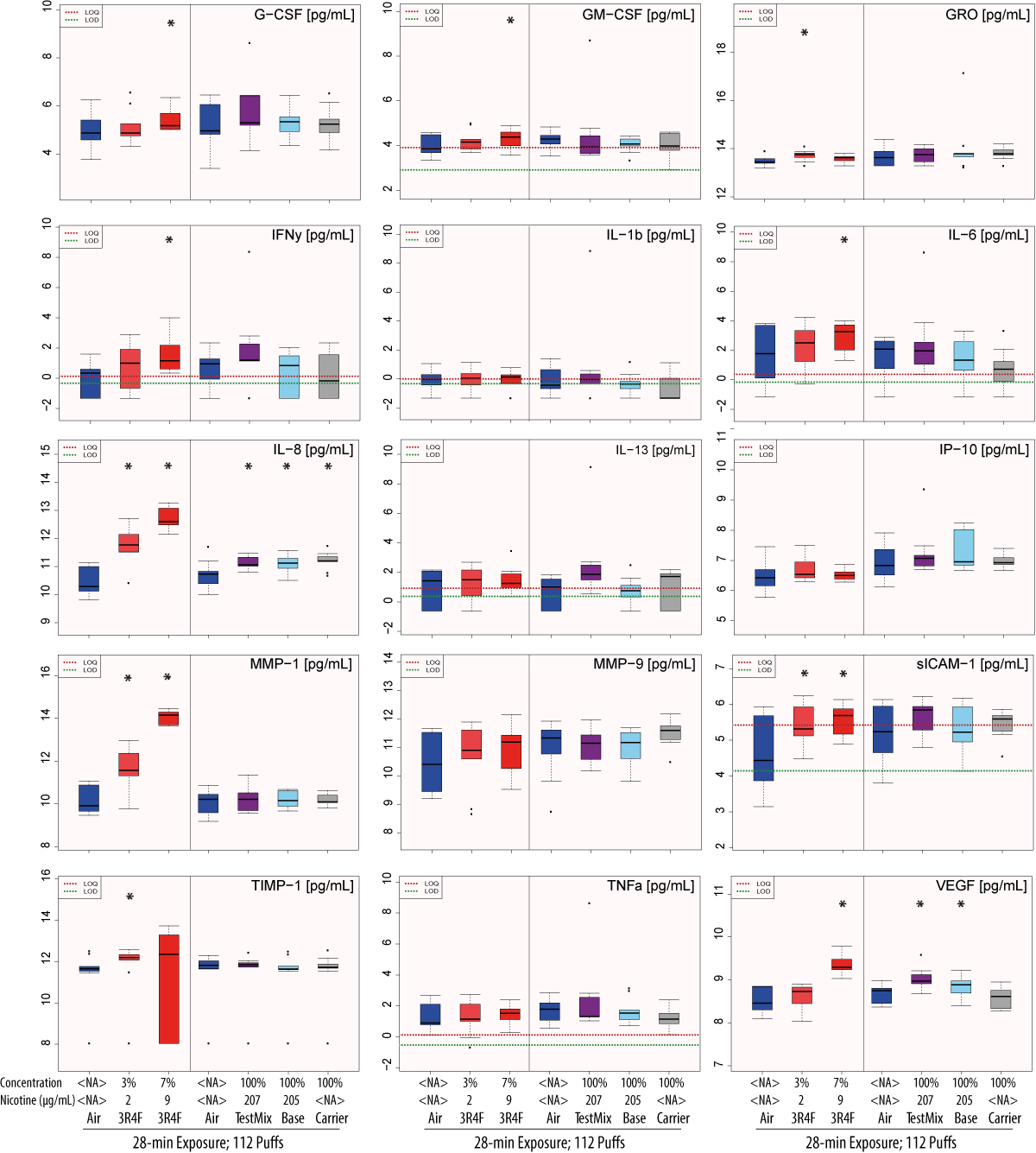


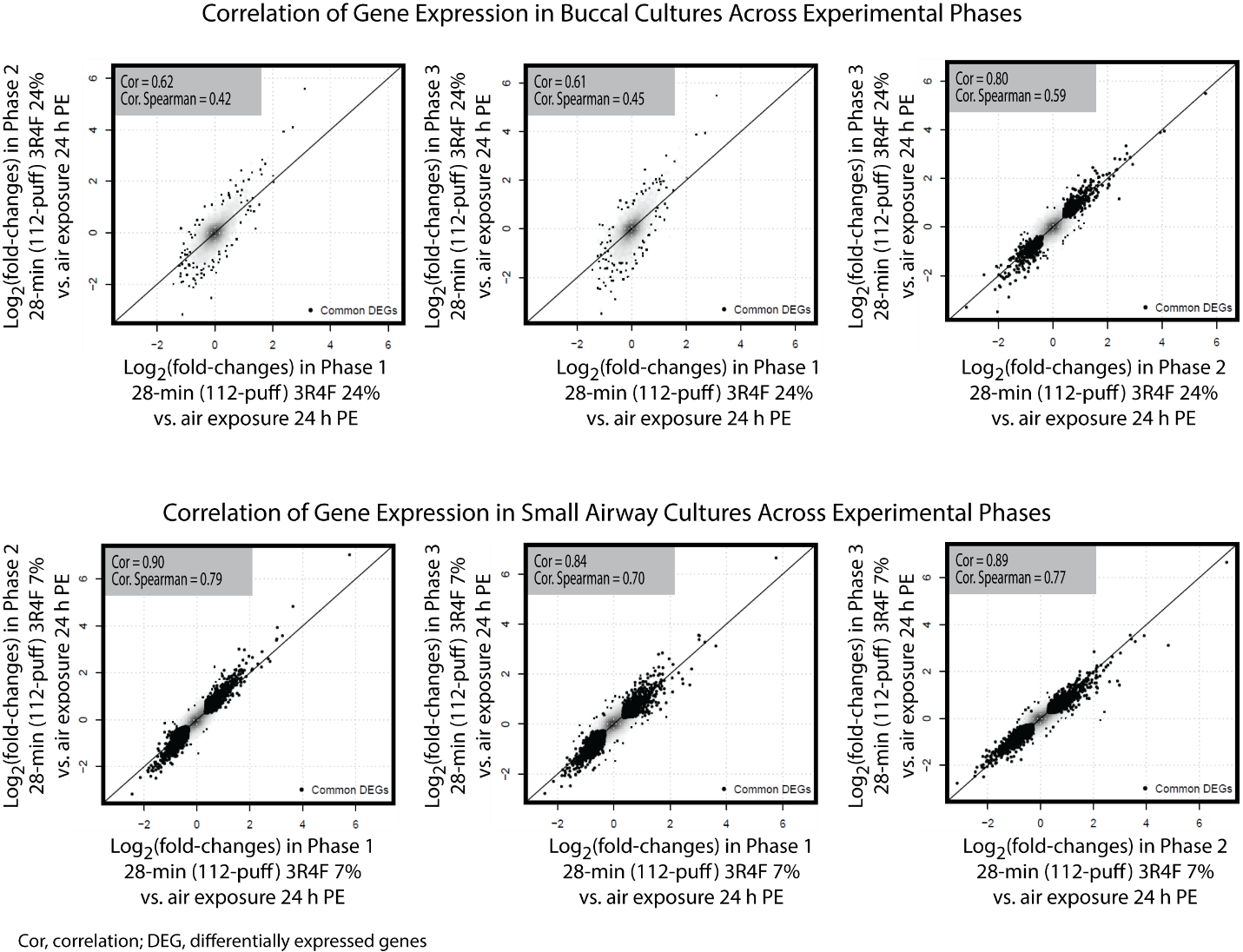


Supplementary Figure 4. Correlation of gene expression changes

The log2(fold-changes) in gene expression in cultures 24 hours post-exposure to 3R4F 24% [*upper panels* for buccal cultures] and to 3R4F CS 7% [*lower panels* for small airway cultures] relative to the expression in cultures exposed to air in a given experimental phase were correlated to the log2(fold-changes) in another experimental phase.


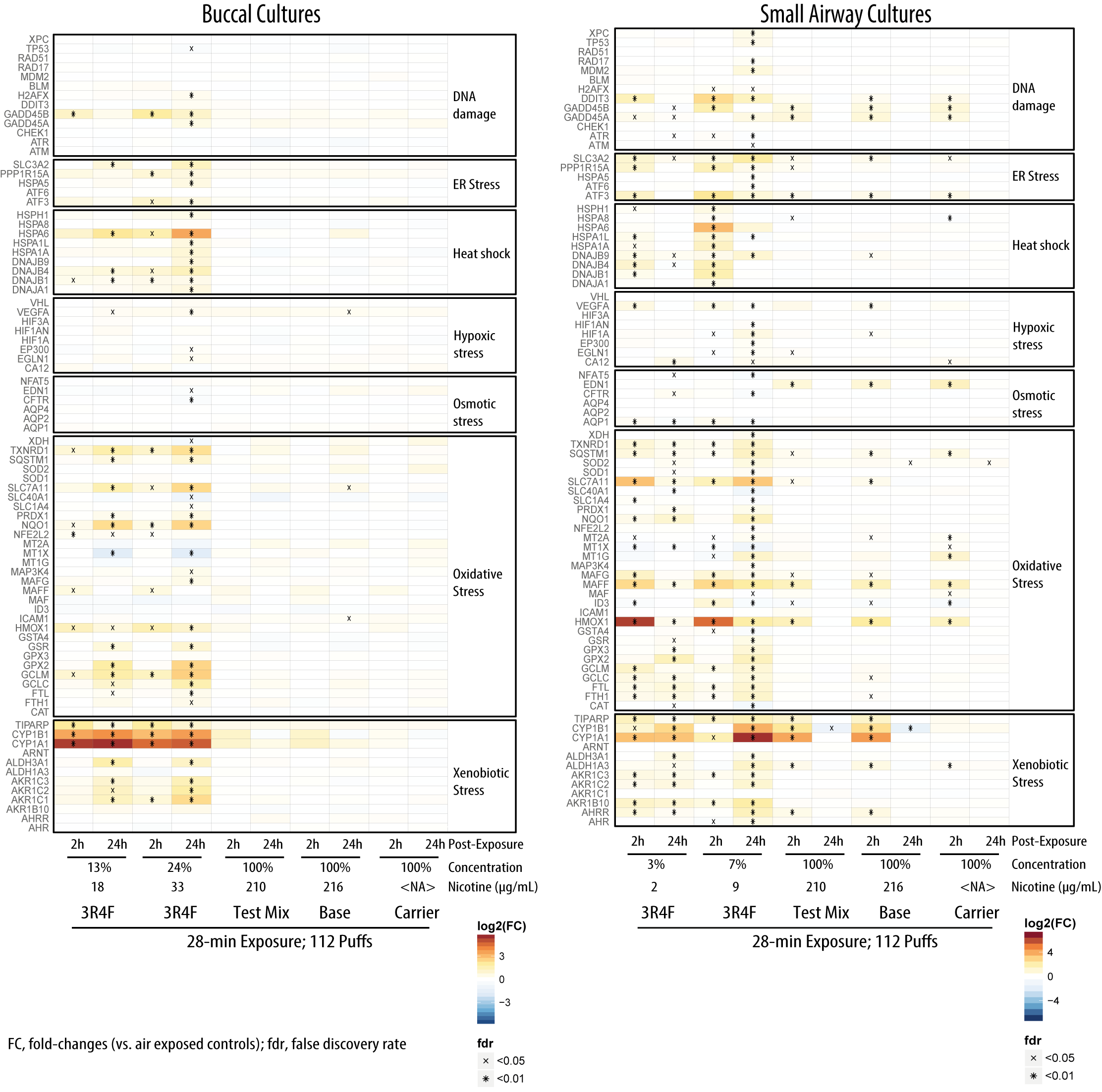


Supplementary Figure 5. Differentially express genes regulating cellular stress response

Heat map showing the differentially express gene expression across the group (compared with the level of the air-exposed samples). Expression values are log_2_(fold-change). Y-axis indicates gene name. Red and blue color intensity indicate upregulation and downregulation, respectively.
